# Sliding mode control with stochastic modeling and mobility interaction for managing epidemic spread in high-population regions

**DOI:** 10.1016/j.parepi.2025.e00439

**Published:** 2025-06-16

**Authors:** Dewi Suhika, Roberd Saragih, Dewi Handayani, Mochamad Apri

**Affiliations:** a Doctoral Program of Mathematics, Faculty of Mathematics and Natural Sciences, Institut Teknologi Bandung, Ganesha, 10, Bandung 40312, Jawa Barat, Indonesia; b Departement of Mathematics, Faculty of Mathematics and Natural Sciences, Institut Teknologi Bandung, Ganesha, 10, Bandung 40312, Jawa Barat, Indonesia; c Mathematics Study Program, Faculty of Sciences, Institut Teknologi Sumatera, Lampung Selatan, 35365, Lampung, Indonesia

**Keywords:** Stochastic epidemiological model, Sliding mode control, Extended Kalman filter, Mobility, Parameter estimation

## Abstract

Managing infectious disease transmission in high-mobility regions is a critical challenge due to dynamic population interactions and elevated transmission risks. This study develops a stochastic epidemiological model to simulate disease spread between two densely populated provinces in Indonesia, Jakarta and West Java. A robust sliding mode control (SMC) framework is proposed and integrated with an Extended Kalman Filter (EKF) to estimate key epidemiological parameters in real time using limited observable data. The proposed framework functions as a theoretical and simulation-based tool to evaluate the potential effects of vaccination and isolation strategies. Although full-state variables are not directly measurable in practice, the EKF allows for the estimation of unobservable parameters, thereby enabling control analysis under uncertainty. Simulation results demonstrate that the SMC strategy significantly reduces infection levels in both provinces, achieving reductions of 84.45 % and 63.94 % in Jakarta, and 98.83 % and 58.35 % in West Java, for the original and Omicron variants, respectively. By incorporating stochasticity, the model captures natural fluctuations and mismatched uncertainties in epidemic progression. This work contributes a conceptual control framework that integrates EKF and SMC for managing stochastic epidemic systems. While the approach is not directly implementable for real-time policymaking, it offers valuable insight into disease dynamics and the potential impact of control strategies under limited observability. These findings support the use of data-driven control simulations for scenario evaluation and policy guidance in complex, uncertain epidemic settings.

## 1 Introduction

In the field of system control, various methods have been developed to manage complex dynamics and uncertainties in real-world applications. One prominent approach is sliding mode control (SMC), recognized for its robustness in handling uncertainties and maintaining stable performance across a wide range of conditions (Behera and Bandyopadhyay, 2017; Shtessel et al., 2014). SMC's primary strength lies in its resilience to external disturbances and model uncertainties without requiring complete information about these disturbances (Khamar and Edrisi, 2018; Van et al., 2017). Typically, conventional SMC is designed to manage uncertainties that can be fully controlled by the input, often referred to as matched uncertainties (Hou et al., 2019).

However, many real systems encounter unmatched uncertainties that cannot be directly addressed by control inputs, especially when the structure of the uncertainty does not align with the system's control pathway. These challenges can reduce system performance or even lead to instability (Xiao et al., 2019). Despite such limitations, SMC remains a compelling solution due to its robustness, although additional strategies are often required to address these uncontrolled components (Coban, 2019; Tran et al., 2020). This makes SMC suitable for applications in domains with significant modeling uncertainty, including infectious disease modeling (Ibarra et al., 2021; Jiao and Shen, 2020; Mehra et al., 2019), where dynamic interactions and unpredictable conditions are prevalent.

In the context of infectious disease modeling, especially during the COVID-19 pandemic, deterministic compartmental models such as SEIR have been widely used. These models divide the population into states such as susceptible $\left(S\right)$, exposed $\left(E\right)$, infected $\left(i\right)$, recovered $\left(R\right)$, and in some cases, vaccinated or quarantined, depending on the model extension. While such models offer useful insights under stable conditions (Qiu et al., 2022; Wang et al., 2022) they assume constant parameters and fail to represent real-world variability. High mobility regions such as Jakarta and West Java have recorded some of the highest COVID-19 and Omicron transmission rates in Indonesia (Djordjevic et al., 2023). Therefore, deterministic assumptions can be overly simplistic due to the complex and fluctuating nature of human movement and interaction.

To address these limitations, stochastic models offer a more realistic alternative by incorporating random disturbances into disease transmission dynamics (Gunasekaran et al., 2023). These models account for fluctuations in contact rates, human behavior, and external factors that influence transmission, enabling a better approximation of real-world disease spread (Selvan and Kumar, 2023). In this study, we extend an existing deterministic model by integrating stochastic components and modeling inter-regional mobility between Jakarta and West Java.

Importantly, we do not assume direct access to all underlying model parameters that influence transmission dynamics, such as effective contact rates and inter-provincial mobility coefficients. Instead, we employ the Extended Kalman Filter (EKF) to estimate these unknown parameters using observable data, specifically the reported number of infections in Jakarta and West Java. This parameter estimation enables a dynamic and data-driven control strategy to adapt to changes in transmission patterns over time.

The estimated parameters are then used within a SMC framework, which applies vaccination and isolation inputs to mitigate the spread of infection. The control design is formulated using integral-proportional (IP) sliding surfaces to enhance robustness under uncertainty. While the proposed framework is not intended for direct deployment as public policy, it serves as a simulation-based tool to evaluate the potential effectiveness of intervention strategies under stochastic and mobility-driven epidemic scenarios. This aligns with ongoing trends in real-time surveillance and adaptive epidemic management.

This paper is structured into five sections. Section 1 introduces the background, motivation, and objectives of the study. Section 2 describes the methods, including the disease transmission model, parameter estimation using EKF, the stochastic model with inter-regional mobility, and the sliding mode control design. Section 3 presents the simulation results and validation of the proposed control framework. Section 4 discusses the findings and implications. Finally, Section 5 concludes the study and summarizes the key contributions.

## 2 Methods

This study develops a stochastic epidemiological model to simulate the spread of COVID-19 across high-mobility regions, focusing on Jakarta and West Java. As the two most densely populated provinces in Indonesia, Jakarta and West Java have consistently recorded the highest rates of COVID-19 and Omicron transmission during the pandemic (Sutrisni et al., 2023). These regions serve as a critical case study for evaluating control strategies due to their dynamic population interactions and high mobility patterns. Building upon the SEIR (Susceptible-Exposed-Infected-Recovered) framework, the model was expanded into $\mathrm{SE}E_{m}\mathrm{VI}i_{m}\mathrm{QR}$ to incorporate vaccination, immunity, and quarantine dynamics (Suhika et al., 2024). In this structure, S denotes the susceptible population, E represents individuals exposed to the original virus $E_{m}$ refers to those exposed to the more transmissible Omicron variant. The compartment V captures vaccinated individuals, while i and $i_{m}$ represent those actively infected with the original and Omicron strains, respectively. The compartment Q contains quarantined individuals, and R includes those who have recovered and gained imunity.

To reflect uncertainty in disease spread due to mobility, testing, and behavior, we introduce a stochastic differential equation (SDE) model of the form:

(1) $$\mathrm{dX}\left(T\right)=f\left(X\left(T\right),u\left(T\right)\right)\mathrm{dt}+G\left(X\left(T\right)\right)\mathrm{dW}\left(T\right)$$

Here, $f\left(X\left(T\right),u\left(T\right)\right)$ encodes the deterministic system dynamics influenced by control inputs $u\left(T\right)$, while $G\left(X\left(T\right)\right)\mathrm{dW}\left(T\right)$ introduces stochastic perturbations modeled as Wiener processes. These stochastic elements allow the model to account for random fluctuations in infection patterns and inter-provincial mobility (Mohammad and Kamrujjaman, 2025).

For clarity and numerical implementation, we first consider the deterministic version of the $\mathrm{SE}E_{m}\mathrm{VI}i_{m}\mathrm{QR}$ model without inter-regional mobility. Each compartment is normalized with respect to the total population N, such that:

$\bar{S}=\frac{S}{N},\bar{E}=\frac{E}{N},{\bar{E}}_{m}=\frac{E_{m}}{N},\bar{V}=\frac{V}{N},\bar{i}=\frac{i}{N},{\bar{i}}_{m}=\frac{i_{m}}{N},\bar{Q}=\frac{Q}{N},$and $\bar{R}=\frac{R}{N},$

These compartments are proportionally consistent and satisfy the condition:

$$\bar{S}+\bar{E}+{\bar{E}}_{m}+\bar{V}+\bar{i}+{\bar{i}}_{m}+\bar{Q}+\bar{R}=1.$$

Based on these definitions, the normalized deterministic system of equations is expressed as follows:

$$\dot{\bar{S}}=b-\beta \bar{S}\bar{i}-\beta_{m}\bar{S}{\bar{i}}_{m}-\alpha \bar{S}-\mu \bar{S}$$

$$\dot{\bar{E}}=\beta \bar{S}\bar{i}+\beta \delta_{1}\bar{V}\bar{i}-\sigma_{1}\bar{E}-\mu \bar{E}$$

(2) $${\dot{\bar{E}}}_{m}=\beta_{m}\bar{S}{\bar{i}}_{m}+\beta_{m}\delta_{2}\bar{V}{\bar{i}}_{m}-\sigma_{2}{\bar{E}}_{m}-\mu {\bar{E}}_{m}$$

$$\dot{\bar{V}}=\alpha \bar{S}-\beta \delta_{1}\bar{V}\bar{i}-\beta_{m}\delta_{2}\bar{V}{\bar{i}}_{m}-\mu \bar{V}$$

$$\dot{\bar{i}}=\sigma_{1}\bar{E}-u_{2}\bar{i}-\gamma_{1}\bar{i}-\mu \bar{i}$$

$${\dot{\bar{i}}}_{m}=\sigma_{2}{\bar{E}}_{m}-u_{2}{\bar{i}}_{m}-\gamma_{2}{\bar{i}}_{m}-\mu {\bar{i}}_{m}$$

$$\dot{\bar{Q}}=u_{2}\bar{i}+u_{2}{\bar{i}}_{m}-\gamma_{3}\bar{Q}-\mu \bar{Q}$$

$$\dot{\bar{R}}=\gamma_{1}\bar{i}+\gamma_{2}{\bar{i}}_{m}+\gamma_{3}\bar{Q}-\mu \bar{R}$$

The initial conditions ensure that all state variables are non-negative at time $T=0,\bar{S}\left(0\right)\geq 0,\bar{E}\left(0\right)\geq 0,{\bar{E}}_{m}\left(0\right)\geq 0,\bar{V}\left(0\right)\geq 0,\bar{i}\left(0\right)\geq 0,{\bar{i}}_{m}\left(0\right)\geq 0,\bar{Q}\left(0\right)\geq 0,\bar{R}\left(0\right)\geq 0.$ A detailed description of the model parameters used in the equations above is provided in Table 1.

**Table 1:** Parameters in model for spread of COVID-19.

Parameters	Definitions
β	Transmission rate of the original virus
$\beta_{m}$	Transmission rate of the omicron virus
α	Vaccination rates
$\delta_{1}$	Antibodies may not have been formed by vaccine recipients against the original virus after 28 days.
$\delta_{2}$	Antibodies may not have formed in vaccine recipients against the Omicron virus after 28 days.
$\sigma_{1}$	The rate of change of individual exposures from E to i
$\sigma_{2}$	The rate of change of individual exposures $E_{m}$ to $i_{m}$
μ	Natural death rate
$\gamma_{1}$	Recovery rate of each compartment i
$\gamma_{2}$	Recovery rate of each compartment $i_{m}$
$\gamma_{3}$	Recovery rate of each compartment Q

To account for inter-regional mobility, the model introduces inter-provincial flow rates that represent the movement of individuals between Jakarta and West Java. This mobility is integrated into the compartmental transitions, modifying the rates of exposure, infection, and recovery. Mobility parameters were derived directly from COVID-19 case data in these provinces, normalized to represent inter-regional flow dynamics without requiring external mobility datasets. The spread of COVID-19 is modeled using stochastic differential equations (SDEs) to incorporate both deterministic dynamics and random fluctuations caused by population mobility and behavioral changes.

To dynamically estimate inter-regional mobility rates, transmission rates, and recovery rates, an Extended Kalman Filter (EKF) was employed. COVID-19 case data from Jakarta and West Java, spanning June 1 to August 31, 2021, were used to estimate parameters. The accuracy of the estimated parameters was evaluated using RRMSE (Relative Root Mean Square Error), calculated based on the observable output $y_{K}=\left[i_{i}\left(T_{K}\right),i_{j}\left(T_{K}\right)\right]$, as follows

(4) $$\text{RRMSE}=\frac{1}{N_{A}}\sum_{K=1}^{N_{A}}\frac{{\left\|y_{K}-{\hat{y}}_{K}\right\|}^{2}}{{\left\|y_{K}\right\|}^{2}}$$

where $N_{A}$ is the total number of observations, $y_{K}$ represents the actual reported infection levels at time $T_{K}$, and ${\hat{y}}_{K}$ denotes the model's predicted output at the same time, based on the parameters estimated by the EKF (Hasan and Nasution, 2022).

Based on the estimated parameters, a sliding mode control strategy was implemented to mitigate the spread of COVID-19 under stochastic and mismatched uncertainties. The control design incorporated an integral-proportional (IP) sliding surface to enhance stability and robustness (Moghadam and Kebriaei, 2020; Sam et al., 2004). The control law consists of an equivalent control component that stabilizes the system and a switching control component that ensures convergence to the sliding surface. The stability of the sliding mode control design was analyzed using Lyapunov theory. A Lyapunov candidate function was selected to demonstrate exponential convergence of the system to the desired trajectory under both deterministic and stochastic uncertainties. The analysis confirmed that the system satisfies the conditions for stability, ensuring robust control performance.

**Fig. 1:**
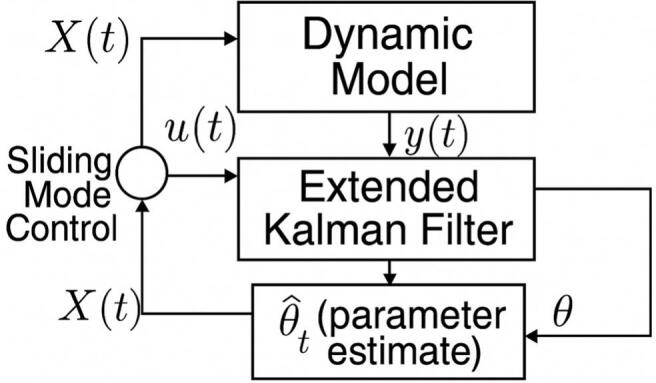
Closed-loop control system combining EKF and sliding mode control.

Fig. 1 illustrates the closed-loop control system developed in this study. The framework integrates an EKF with a SMC strategy based on integral-proportional (IP) sliding surfaces. The stochastic epidemiological model, denoted by the state vector $X\left(T\right)$, evolves dynamically under uncertain inter-regional mobility between Jakarta and West Java. In this configuration, the EKF processes observed infection data specifically $i_{i}\left(T\right)$ and $i_{j}\left(T\right)$ to estimate time-varying epidemiological parameters ${\hat{\theta }}_{T}$, such as transmission rates and mobility coefficients, which are not directly measurable. The true (but unknown) parameter vector is denoted by θ, and the diagram reflects how the EKF generates ${\hat{\theta }}_{T}$ as its best estimate of θ.

These parameter estimates are then forwarded to the SMC module. The control law is constructed directly from the system state $X\left(T\right)$ using an IP-based sliding surface formulation, without the need for an explicit reference signal. The resulting control signals $u_{1i}\left(T\right),u_{2i}\left(T\right),u_{1j}\left(T\right),u_{2j}\left(T\right)$ represent vaccination and isolation interventions in each region. These inputs are applied to the dynamic model, which evolves under both deterministic and stochastic influences. The EKF continually updates the parameter estimates based on the model outputs $y\left(T\right)=\left[i_{i}\left(T\right),i_{j}\left(T\right)\right]$, forming a complete feedback loop that enables adaptive control in the presence of system uncertainties and stochastic perturbations.

## 3 Results

### 3.1 Disease transmission model

**Fig. 2:**
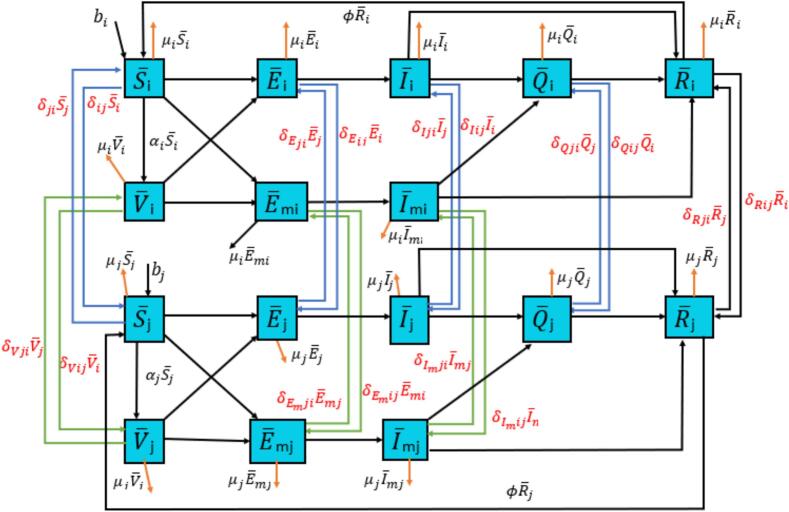
Diagram of COVID-19 transmission with mobility aspect.

In this section, the COVID-19 model is further developed by integrating inter-regional mobility factors to capture disease transmission dynamics more realistically. This model provides insights into the impact of human movement on transmission dynamics by simulating how individuals in one region may become infected, recover, or travel to other regions, thus influencing overall transmission rates. Fig. 2 illustrates the flow of individuals and inter-regional interactions, highlighting how mobility affects disease spread and the effectiveness of regional intervention policies. This mobility-accommodating model enables a deeper understanding of intervention strategies, such as travel restrictions or regional quarantines, which play a crucial role in controlling the spread of COVID-19. Based on this concept, the mathematical formulation for the Omicron variant, incorporating mobility aspects, is presented in system (5).

Within cluster-i, the population is divided into eight compartments: the susceptible subpopulation $\left({\bar{S}}_{i}\right)$, individuals exposed to the original virus $\left({\bar{E}}_{i}\right)$, individuals exposed to the Omicron variant $\left({\bar{E}}_{\mathrm{mi}}\right)$, vaccinated individuals $\left({\bar{V}}_{i}\right)$, individuals infected with the original virus$\left({\bar{i}}_{®i}\right)$, individuals infected with Omicron $\left({\bar{i}}_{\mathrm{mi}}\right)$, hospitalized individuals $\left({\bar{Q}}_{i}\right)$, and individuals who have recovered or are immune $\left({\bar{R}}_{i}\right)$. Together, these compartments form the total population in cluster i, denoted as $N_{i}$. Similarly, cluster-j has a comparable structure with eight compartments: the susceptible subpopulation $\left({\bar{S}}_{j}\right)$, individuals exposed to the original virus $\left({\bar{E}}_{j}\right)$, individuals exposed to Omicron $\left({\bar{E}}_{\mathrm{mj}}\right)$, vaccinated individuals $\left({\bar{V}}_{j}\right)$, individuals infected with the original virus $\left({\bar{i}}_{j}\right)$, individuals infected with Omicron $\left({\bar{i}}_{\mathrm{mj}}\right)$, hospitalized individuals $\left({\bar{Q}}_{j}\right)$, and individuals who have recovered or are immune $\left({\bar{R}}_{j}\right)$, forming the total population $N_{j}$.

$${\dot{\bar{S}}}_{i}=b_{i}-\beta_{i}{\bar{S}}_{i}{\bar{i}}_{i}-\beta_{\mathrm{mi}}{\bar{S}}_{i}{\bar{i}}_{\mathrm{mi}}-\left(1+u_{1i}\left(T\right)\right)\alpha_{i}{\bar{S}}_{i}-\mu_{i}{\bar{S}}_{i}+\delta_{\mathrm{ji}}{\bar{S}}_{j}-\delta_{\mathrm{ij}}{\bar{S}}_{i}+\phi {\bar{R}}_{i}$$

$${\dot{\bar{E}}}_{i}=\beta_{i}{\bar{S}}_{i}{\bar{i}}_{i}+\beta_{i}\delta_{1i}{\bar{V}}_{i}{\bar{i}}_{i}-\sigma_{1i}{\bar{E}}_{i}-\mu_{i}{\bar{E}}_{i}+{\delta_{E}}_{\mathrm{ji}}{\bar{E}}_{j}-{\delta_{E}}_{\mathrm{ij}}{\bar{E}}_{i}$$

$${\dot{\bar{E}}}_{\mathrm{mi}}=\beta_{\mathrm{mi}}{\bar{S}}_{i}{\bar{i}}_{\mathrm{mi}}+\beta_{\mathrm{mi}}\delta_{2i}{\bar{V}}_{i}{\bar{i}}_{\mathrm{mi}}-\sigma_{2i}{\bar{E}}_{\mathrm{mi}}-\mu_{i}{\bar{E}}_{\mathrm{mi}}+\delta_{E_{m}\mathrm{ji}}{\bar{E}}_{\mathrm{mj}}-\delta_{E_{m}\mathrm{ij}}{\bar{E}}_{\mathrm{mi}}$$

$${\dot{\bar{V}}}_{i}=\alpha_{i}{\bar{S}}_{i}\left(1+u_{1i}\left(T\right)\right)-\beta_{i}\delta_{1i}{\bar{V}}_{i}{\bar{i}}_{i}-\beta_{\mathrm{mi}}\delta_{2i}{\bar{V}}_{i}{\bar{i}}_{\mathrm{mi}}-\mu_{i}{\bar{V}}_{i}+\delta_{\mathrm{Vji}}{\bar{V}}_{j}-\delta_{\mathrm{Vij}}{\bar{V}}_{i}$$

$${\dot{\bar{i}}}_{i}=\sigma_{1i}{\bar{E}}_{i}-u_{2i}\left(T\right){\bar{i}}_{i}-\gamma_{1i}{\bar{i}}_{i}-\mu_{i}{\bar{i}}_{i}+\delta_{\mathrm{Iji}}{\bar{i}}_{j}-\delta_{\mathrm{Iij}}{\bar{i}}_{i}$$

$${\dot{\bar{i}}}_{\mathrm{mi}}=\sigma_{2i}{\bar{E}}_{\mathrm{mi}}-u_{2i}\left(T\right){\bar{i}}_{\mathrm{mi}}-\gamma_{2i}{\bar{i}}_{\mathrm{mi}}-\mu_{i}{\bar{i}}_{\mathrm{mi}}+\delta_{i_{m}\mathrm{ji}}{\bar{i}}_{\mathrm{mj}}-\delta_{i_{m}\mathrm{ij}}{\bar{i}}_{\mathrm{mi}}$$

$${\dot{\bar{Q}}}_{i}=u_{2i}\left(T\right){\bar{i}}_{i}+u_{2i}\left(T\right){\bar{i}}_{\mathrm{mi}}-\gamma_{3i}{\bar{Q}}_{i}-\mu_{i}{\bar{Q}}_{i}+\delta_{\mathrm{Qji}}{\bar{Q}}_{j}-\delta_{\mathrm{Qij}}{\bar{Q}}_{i}$$

(5) $${\dot{\bar{R}}}_{i}=\gamma_{1i}{\bar{i}}_{i}+\gamma_{2i}{\bar{i}}_{\mathrm{mi}}+\gamma_{3i}{\bar{Q}}_{i}-\mu_{i}{\bar{R}}_{i}-S\phi_{S}{\bar{R}}_{i}+\delta_{\mathrm{Rji}}{\bar{R}}_{j}-\delta_{\mathrm{Rij}}{\bar{R}}_{i}$$

$${\dot{\bar{S}}}_{j}=b_{j}-\beta_{j}{\bar{S}}_{j}{\bar{i}}_{j}-\beta_{\mathrm{mj}}{\bar{S}}_{j}{\bar{i}}_{\mathrm{mj}}-\left(1+u_{1j}\left(T\right)\right)\alpha_{j}{\bar{S}}_{j}-\mu_{j}{\bar{S}}_{j}+\delta_{\mathrm{ij}}{\bar{S}}_{i}-\delta_{\mathrm{ji}}{\bar{S}}_{j}+\phi {\bar{R}}_{j}$$

$${\dot{\bar{E}}}_{j}=\beta_{j}{\bar{S}}_{j}{\bar{i}}_{j}+\beta_{j}\delta_{1j}{\bar{V}}_{j}{\bar{i}}_{j}-\sigma_{1j}{\bar{E}}_{j}-\mu_{j}{\bar{E}}_{j}+{\delta_{E}}_{\mathrm{ij}}{\bar{E}}_{i}-{\delta_{E}}_{\mathrm{ji}}{\bar{E}}_{j}$$

$${\dot{\bar{E}}}_{\mathrm{mj}}=\beta_{\mathrm{mj}}{\bar{S}}_{j}{\bar{i}}_{\mathrm{mj}}+\beta_{\mathrm{mj}}\delta_{2j}{\bar{V}}_{j}{\bar{i}}_{\mathrm{mj}}-\sigma_{2j}{\bar{E}}_{\mathrm{mj}}-\mu_{j}{\bar{E}}_{\mathrm{mj}}+\delta_{E_{m}\mathrm{ij}}{\bar{E}}_{\mathrm{mi}}-\delta_{E_{m}\mathrm{ji}}{\bar{E}}_{\mathrm{mj}}$$

$${\dot{\bar{V}}}_{j}=\alpha_{j}{\bar{S}}_{j}\left(1+u_{1j}\left(T\right)\right)-\beta_{j}\delta_{1j}{\bar{V}}_{j}{\bar{i}}_{j}-\beta_{\mathrm{mj}}\delta_{2j}{\bar{V}}_{j}{\bar{i}}_{\mathrm{mj}}-\mu_{j}{\bar{V}}_{j}-\delta_{\mathrm{Vji}}{\bar{V}}_{j}+\delta_{\mathrm{Vij}}{\bar{V}}_{i}$$

$${\dot{\bar{i}}}_{j}=\sigma_{1j}{\bar{E}}_{j}-u_{2j}\left(T\right){\bar{i}}_{j}-\gamma_{1j}{\bar{i}}_{j}-\mu_{j}{\bar{i}}_{j}+\delta_{\mathrm{Iij}}{\bar{i}}_{i}-\delta_{\mathrm{Iji}}{\bar{i}}_{j}$$

$${\dot{\bar{i}}}_{\mathrm{mj}}=\sigma_{2j}{\bar{E}}_{\mathrm{mj}}-u_{2j}\left(T\right){\bar{i}}_{\mathrm{mj}}-\gamma_{2j}{\bar{i}}_{\mathrm{mj}}-\mu_{j}{\bar{i}}_{\mathrm{mj}}+\delta_{i_{m}\mathrm{ij}}{\bar{i}}_{\mathrm{mi}}-\delta_{i_{m}\mathrm{ji}}{\bar{i}}_{\mathrm{mj}}$$

$${\dot{\bar{Q}}}_{j}=u_{2j}\left(T\right){\bar{i}}_{j}+u_{2j}\left(T\right){\bar{i}}_{\mathrm{mj}}-\gamma_{3j}{\bar{Q}}_{j}-\mu_{j}{\bar{Q}}_{j}+\delta_{\mathrm{Qij}}{\bar{Q}}_{i}-\delta_{\mathrm{Qji}}{\bar{Q}}_{j}$$

$${\dot{\bar{R}}}_{j}=\gamma_{1j}{\bar{i}}_{j}+\gamma_{2j}{\bar{i}}_{\mathrm{mj}}+\gamma_{3j}{\bar{Q}}_{j}-\mu_{j}{\bar{R}}_{j}-\phi_{S}{\bar{R}}_{j}+\delta_{\mathrm{Rij}}{\bar{R}}_{i}-\delta_{\mathrm{Rji}}{\bar{R}}_{j}$$

A detailed description of each compartment is provided in Table 2, offering a comprehensive overview of the model structure and the role of mobility in influencing COVID-19 spread.

**Table 2:** Parameters in the mobility model for spread of COVID-19.

Parameters	Definitions	Values	Sources
$B_{i}$	Birth rate in cluster-i	$\frac{1}{74.8\times 365}$	(BPS, 2021)
$\mu_{i}$	Natural death rate in cluster-i	$\frac{1}{74.8\times 365}$	(BPS, 2021)
$\beta_{i}$	Original virus infection rate in cluster-i	0.1247	Estimated
$\beta_{\mathrm{mi}}$	Omicron infection rate in the cluster-i	0.2658	Estimated
$\alpha_{i}$	Vaccination rate in cluster-i	0.0016	Estimated
$\delta_{\mathrm{ji}}$	The probability of susceptible individuals moving from cluster-ito cluster-j	0.4420	Estimated
$\delta_{\mathrm{ij}}$	The probability of susceptible individuals moving from cluster-j to cluster-i	0.0048	Estimated
$\sigma_{1i}$	The rate of change of exposed individuals becoming infected with the original virus in cluster-i	0.0358	Estimated
$\sigma_{2i}$	The rate of change of exposed individuals becoming infected with Omicron in cluster-i	0.2363	Estimated
$\delta_{1i}$	The probability that a vaccine recipient in cluster-i does not form antibodies against the original virus after 28 days	0.05	(Kim et al., 2022)
$\delta_{2i}$	The probability that vaccine recipients in cluster-i do not develop antibodies against Omicron after 8 days	0.33	(Chi et al., 2022)
${\delta_{E}}_{\mathrm{ij}}$	The probability of movement of individuals exposed to the original virus from cluster-i to cluster-j	0.1015	Estimated
${\delta_{E}}_{\mathrm{ji}}$	The probability of exposed individuals moving from cluster-j to cluster-i	0.2929	Estimated
$\delta_{E_{m}\mathrm{ij}}$	The probability of exposed individuals moving from cluster-i to cluster-j	0.5885	Estimated
$\delta_{E_{m}\mathrm{ji}}$	The probability of exposed individuals moving from cluster-j to cluster-i	0.5050	Estimated
$\delta_{\mathrm{Vij}}$	The probability of vaccinated individuals moving from cluster-i to cluster-j	0.5317	Estimated
$\delta_{\mathrm{Vji}}$	The probability of vaccinated individuals moving from cluster-j to cluster-i	0.5995	Estimated
ϕ	The rate of immunity loss and return to the $\bar{S}$ compartment	1/180	(BPS, 2021)
$\gamma_{1i}$	The recovery rate of individuals infected with the original virus in cluster-i	0.0002	Estimated
$\delta_{\mathrm{Iij}}$	The probability of infected individuals moving from cluster-i to cluster-j	0.0201	Estimated
$\delta_{\mathrm{Iji}}$	The probability of infected individuals moving from cluster-j to cluster-i	0.0154	Estimated
$\gamma_{2i}$	The recovery rate of individuals Q in cluster-i	0.6083	Estimated
$\delta_{i_{m}\mathrm{ij}}$	The probability of an infected person with Omicron moving from cluster-i to cluster-j	0.5533	Estimated
$\delta_{i_{m}\mathrm{ji}}$	The probability of an infected person with Omicron moving from cluster-j to cluster-i	0.4948	Estimated
$\gamma_{3i}$	The recovery rate of individuals infected with Omicron in cluster-i	0.5471	Estimated
$\delta_{\mathrm{Qij}}$	The probability of Q moving from cluster-i to cluster-j	0.0039	Estimated
$\delta_{\mathrm{Qji}}$	The probability of Q moving from cluster-j to cluster-i	0.6497	Estimated
$\delta_{\mathrm{Rij}}$	The probability of recovered individuals moving from cluster-i to cluster-j	0.2342	Estimated
$\delta_{\mathrm{Rji}}$	The probability of recovered individuals moving from cluster-j to cluster-i	0.7280	Estimated
$B_{j}$	Birth rate in cluster-j	$\frac{1}{75\times 365}$	(BPS, 2021)
$\beta_{j}$	Original virus infection rate in cluster-j	0.6319	Estimated
$\beta_{\mathrm{mj}}$	Omicron infection rate in the cluster-j	0.4715	Estimated
$\alpha_{j}$	Vaccination rate in cluster-j	0.0565	Estimated
$\sigma_{1j}$	The rate of change of exposed individuals becoming infected with the original virus in cluster-j	0.0145	Estimated
$\sigma_{2j}$	The rate of change of exposed individuals becoming infected with Omicron in cluster-j	0.5358	Estimated
$\delta_{1j}$	The probability that a vaccine recipient in cluster-j does not form antibodies against the original virus after 28 days	0.05	(Kim et al., 2022)
$\delta_{2j}$	The probability that vaccine recipients in cluster-j do not develop antibodies against Omicron after 28 days	0.33	(Chi et al., 2022)
$\gamma_{1j}$	The recovery rate of individuals infected with the original virus in cluster-j	0.0001	Estimated
$\gamma_{2j}$	The recovery rate of individuals Q in cluster-j	0.5217	Estimated
$\gamma_{3j}$	The recovery rate of individuals infected with Omicron in cluster-j	0.3696	Estimated
$u_{2i}$	Isolation rate in cluster-i	0.0006	Estimated
$u_{2j}$	Isolation rate in cluster-j	0.0001	Estimated

In the model represented by eq. (5), control inputs are applied to manage the spread of COVID-19 through vaccination and isolation measures in two regions, namely Jakarta and West Java. The control input $u_{1i}$ denotes the vaccination rate in Jakarta, measured as a proportion of the population vaccinated per day (unit: 1/day), while $u_{2i}$ represents the isolation rate, indicating the fraction of the infected population isolated per day. Similarly, $u_{1j}$ and $u_{2j}$ correspond to the vaccination and isolation rates, respectively, in West Java. These control inputs are constrained by practical considerations: vaccination rates are limited by vaccine availability and distribution capacity, while isolation rates are influenced by social and logistical feasibility. Although these control inputs are not meant to represent directly implementable public health policies, they provide an idealized control framework to evaluate the potential impact of vaccination and isolation strategies under uncertainty. By incorporating these controls into a data-driven simulation model, the study assesses how targeted interventions may reduce the susceptible population, lower transmission rates, and stabilize infection dynamics in the high-mobility regions of Jakarta and West Java.

### 3.2 Parameter estimation

**Fig. 3:**
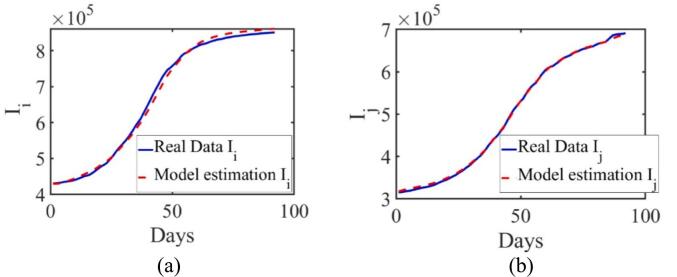
Comparison of the simulation model (blue line) with data on individual COVID-19 infection cases in cluster-i (Jakarta) and cluster-j (West Java). (For interpretation of the references to colour in this figure legend, the reader is referred to the web version of this article.)

In this study, cumulative COVID-19 case data from June 1 to August 31, 2021, were used for parameter estimation. Jakarta was observed as cluster-i and West Java as cluster-j. A total of 34 model parameters were estimated using the EKF, as summarized in Table 2. Fig. 3 presents the validation results, comparing real data with EKF-based estimation outputs for the infected populations $i_{i}$ and $i_{j}$. In both graphs, the solid blue lines represent actual reported data, while the dashed red lines represent the estimated trajectories generated by the model. The EKF estimates these states based on available observable data (such as positive case counts), even though the full internal epidemiological states are not directly measurable. This allows the model to operate under realistic data limitations.

To evaluate the accuracy of the parameter estimation, the RRMSE was applied (Hasan and Nasution, 2022). This metric quantifies the deviation between the model estimations and the actual observations. For the infected population $i_{i}$, the RRMSE was calculated as 0.0296 (2.96 %), while for $i_{j}$, the RRMSE was 0.0125 (1.25 %). These low RRMSE values indicate a high level of accuracy in capturing the observed dynamics in both clusters. Overall, EKF demonstrates the capability to infer key unmeasured epidemiological states from limited observable data, even in the presence of noise. This strengthens the credibility of the model for real-time simulation and supports the design of responsive and adaptive public health control strategies.

### 3.3 Stochastic model with mobility aspects

To incorporate stochastic uncertainties that arise from unpredictable factors such as behavioral changes and inter-regional mobility, the deterministic model in eq. (5) is extended into a stochastic framework. This modification enables the model to more accurately capture real-world variability in disease transmission.

Let $X\left(T\right)={\left[{\bar{S}}_{i},{\bar{E}}_{i},{\bar{E}}_{\mathrm{mi}},{\bar{V}}_{i},{\bar{i}}_{i},{\bar{i}}_{\mathrm{mi}},{\bar{Q}}_{i},{\bar{R}}_{i},{\bar{S}}_{j},{\bar{E}}_{j},{\bar{E}}_{\mathrm{mj}},{\bar{V}}_{j},{\bar{i}}_{j},{\bar{i}}_{\mathrm{mj}},{\bar{Q}}_{j},{\bar{R}}_{j}\right]}^{T}$ denote the state vector of the system, where each component represents the normalized proportion of the population in each epidemiological compartment at time T. Here, subscript i refers to Jakarta (cluster-i) and.

j to West Java (cluster-j). The initial condition is given by:

$$X\left(0\right)=[{\bar{S}}_{i}\left(0\right),{\bar{E}}_{i}\left(0\right),{\bar{E}}_{\mathrm{mi}}\left(0\right),{\bar{V}}_{i}\left(0\right),{\bar{i}}_{i}\left(0\right),{\bar{i}}_{\mathrm{mi}}\left(0\right),{\bar{Q}}_{i}\left(0\right),{\bar{R}}_{i}\left(0\right),{\bar{S}}_{j}\left(0\right),{\bar{E}}_{j}\left(0\right),$$

$${\bar{E}}_{\mathrm{mj}}\left(0\right),{\bar{V}}_{j}\left(0\right),{\bar{i}}_{j}\left(0\right),{\bar{i}}_{\mathrm{mj}}\left(0\right),{\bar{Q}}_{j}\left(0\right),{\bar{R}}_{j}\left(0\right)]{}^{T}$$

Each state variable $X_{j},j=1,\ldots ,16$, is modeled as a stochastic process. The stochastic evolution of each state is represented by

$$∆X_{j}=X_{j}\left(T+∆T\right)-X_{j}\left(T\right)$$

which accounts for both deterministic dynamics and stochastic disturbances. The incorporation of randomness enables the model to reflect uncertainty in transmission and recovery rates caused by environmental and behavioral variability.

The disturbance weighting matrix $G_{G}$, defined as $\mathrm{Gg}=\frac{E\left[\left(∆X\right){\left(∆X\right)}^{T}\right]}{∆T}$, represents the weighting matrix for disturbances. The detailed procedure for obtaining $G_{G}$ is provided in the (supplementary information).

$$\mathrm{Gg}=\left(\begin{array}{cc} A_{11} & A_{22}\\ A_{22} & A_{33} \end{array}\right)$$

with

$$A_{11}=\left(\begin{array}{cccccccc} G_{1} & -G_{2} & -G_{3} & -G_{4} & 0 & 0 & 0 & -G_{5}\\ -G_{2} & G_{7} & 0 & -G_{8} & -G_{10} & 0 & 0 & 0\\ -G_{3} & 0 & G_{9} & -G_{14} & 0 & -G_{15} & 0 & 0\\ -G_{4} & -G_{8} & -G_{14} & G_{11} & 0 & 0 & 0 & 0\\ 0 & -G_{10} & 0 & 0 & G_{13} & 0 & -G_{22} & -G_{23}\\ 0 & 0 & -G_{15} & 0 & 0 & G_{17} & -G_{27} & -G_{28}\\ 0 & 0 & 0 & 0 & -G_{22} & -G_{27} & G_{19} & -G_{32}\\ -G_{5} & 0 & 0 & 0 & -G_{23} & -G_{28} & -G_{32} & G_{21} \end{array}\right)$$

$$A_{22}=\left(\begin{array}{cccccccc} -G_{6} & 0 & 0 & 0 & 0 & 0 & 0 & 0\\ 0 & -G_{12} & 0 & 0 & 0 & 0 & 0 & 0\\ 0 & 0 & -G_{16} & 0 & 0 & 0 & 0 & 0\\ 0 & 0 & 0 & -G_{18} & 0 & 0 & 0 & 0\\ 0 & 0 & 0 & 0 & -G_{20} & 0 & 0 & 0\\ 0 & 0 & 0 & 0 & 0 & -G_{24} & 0 & 0\\ 0 & 0 & 0 & 0 & 0 & 0 & -G_{26} & 0\\ 0 & 0 & 0 & 0 & 0 & 0 & 0 & -G_{30} \end{array}\right)$$

$$A_{33}=\left(\begin{array}{cccccccc} G_{25} & -G_{40} & -G_{41} & -G_{42} & 0 & 0 & 0 & -G_{44}\\ -G_{40} & G_{29} & 0 & -G_{45} & -G_{46} & 0 & 0 & 0\\ -G_{41} & 0 & G_{31} & -G_{48} & 0 & -G_{49} & 0 & 0\\ -G_{42} & -G_{45} & -G_{48} & G_{33} & 0 & 0 & 0 & 0\\ 0 & -G_{46} & 0 & 0 & G_{34} & 0 & -G_{52} & -G_{53}\\ 0 & 0 & -G_{49} & 0 & 0 & G_{35} & -G_{55} & -G_{56}\\ 0 & 0 & 0 & 0 & -G_{52} & -G_{55} & G_{36} & -G_{58}\\ -G_{44} & 0 & 0 & 0 & -G_{53} & -G_{56} & -G_{58} & G_{37} \end{array}\right)$$

The determinant and trace of matrix $G_{G}$ are non-negative, $\text{det}\left(G_{G}\right)\geq 0$ and $\text{Trace}\left(G_{G}\right)\geq 0$. Consequently, all eigenvalues of matrix $G_{G}$ are real and non-negative. This matrix can be decomposed using an orthogonal matrix P because $G_{G}$ is a positive semi-definite and symmetric matrix, resulting in $G_{G}=P^{T}\mathrm{DP}$, where P is an orthogonal matrix $\left(PP^{T}=P^{T}P=i\right)$ and $D=\mathrm{Diag}\left(\lambda_{j}\right),j=1,\ldots ,16$. The covariance matrix $\sigma \left(∆X\right)\left(∆X\right)\approx E\left(\left[∆X\right]{\left[∆X\right]}^{T}\right)$ has a unique positive semi-definite square root S, such that $S=P^{T}\sqrt{D}P,$and $S^{2}=\mathrm{Gg}=SS^{T}$, thus $S=\sqrt{\mathrm{Gg}}$. However, there is no analytical method to obtain the square root of a positive semi-definite N×N, matrix for N≥2, so computational methods are often used. The next step is to find the Cholesky factorization of matrix Gg. In this case, matrix S is not shown explicitly but is computed numerically to satisfy $SS^{T}=\mathrm{Gg}$.

In the final stage, it is assumed that $∆X\left(T\right)∼N\left(\mu ∆T,\mathrm{Gg}∆T\right)$, so the stochastic model can be written as in eq. (6). With $SS^{T}=\mathrm{Gg}$ and $w\left(T\right)={\left(w_{1},w_{2},w_{3},w_{4},\ldots ,w_{16}\right)}^{T}$ as a vector of independent Wiener processes, $S\left(X\left(T\right),T\right)$ represents the covariance matrix (often referred to as the volatility matrix) that links changes in variable X with changes in the vector of independent Wiener processes $\left(\mathrm{dW}\left(T\right)\right)$. Based on the deterministic model adapted from eq. (5), the stochastic COVID-19 model can be expressed as:

(6) $$\mathrm{dX}\left(T\right)=\left(\mathrm{AX}\left(T\right)+B_{1}u_{1i}\left(T\right)+B_{2}u_{2i}\left(T\right)+B_{3}u_{1j}\left(T\right)+B_{4}u_{2j}\left(T\right)+f\left(T\right)\right)\mathrm{dt}+S\left(X\left(T\right)\right)\mathrm{dW}\left(T\right).$$

with $\mathrm{dW}\left(T\right)$ being the Wiener process vector (Gaussian white noise), $f\left(X\right)$ is a function representing uncertainty in a system experiencing a mismatched condition. A mismatched condition occurs when the rank of the matrix $\left[B_{1}\left|B_{2}\right|B_{3}|B_{4}|f\left(T\right)\right]$ is not equal to the rank of the matrix $\left[B_{1}\left|B_{2}\right|B_{3}|B_{4}\right]$. This means that the uncertainty $f\left(T\right)$ cannot be fully controlled by the available control inputs. It is assumed that there exists a known positive constant τ such as $\left\|f\left(T\right)\right\|\leq \tau$. This provides an upper bound for $f\left(T\right)$, ensuring that the uncertainty remains within controllable limits. Consequently, this model represents a stochastic model that accounts for multiplicative stochastic disturbances in the system parameters as well as mismatched uncertainty.

$$B_{1}=\left(\begin{array}{c} -\alpha_{i}{\bar{S}}_{i}\\ 0\\ 0\\ \alpha_{i}{\bar{S}}_{i}\\ 0\\ 0\\ 0\\ 0\\ 0\\ 0\\ 0\\ 0\\ 0\\ 0\\ 0\\ 0 \end{array}\right),B_{2}=\left(\begin{array}{c} 0\\ 0\\ 0\\ 0\\ -i_{i}\\ -i_{\mathrm{mi}}\\ i_{i}+i_{\mathrm{mi}}\\ 0\\ 0\\ 0\\ 0\\ 0\\ 0\\ 0\\ 0\\ 0 \end{array}\right),B_{3}=\left(\begin{array}{c} 0\\ 0\\ 0\\ 0\\ 0\\ 0\\ 0\\ 0\\ -\alpha_{j}S_{j}\\ 0\\ 0\\ \alpha_{j}S_{j}\\ 0\\ 0\\ 0\\ 0 \end{array}\right),B_{4}=\left(\begin{array}{c} 0\\ 0\\ 0\\ 0\\ 0\\ 0\\ 0\\ 0\\ 0\\ 0\\ 0\\ 0\\ -i_{j}\\ -i_{\mathrm{mj}}\\ i_{j}+i_{\mathrm{mj}}\\ 0 \end{array}\right)\;\text{and}$$

$$A=\left(\begin{array}{c} B_{i}-\beta_{i}{\bar{S}}_{i}{\bar{i}}_{i}-\beta_{\mathrm{mi}}{\bar{S}}_{i}{\bar{i}}_{\mathrm{mi}}-\alpha_{i}{\bar{S}}_{i}-\mu_{i}{\bar{S}}_{i}+\delta_{\mathrm{ji}}{\bar{S}}_{j}-\delta_{\mathrm{ij}}{\bar{S}}_{i}+\phi_{S}{\bar{R}}_{i}\\ \beta_{i}{\bar{S}}_{i}{\bar{i}}_{i}+\beta_{i}\delta_{1i}{\bar{V}}_{i}{\bar{i}}_{i}-\sigma_{1i}{\bar{E}}_{i}-\mu_{i}{\bar{E}}_{i}+{\delta_{E}}_{\mathrm{ji}}{\bar{E}}_{j}-{\delta_{E}}_{\mathrm{ij}}{\bar{E}}_{i}\\ \beta_{\mathrm{mi}}{\bar{S}}_{i}{\bar{i}}_{\mathrm{mi}}+\beta_{\mathrm{mi}}\delta_{2i}{\bar{V}}_{i}{\bar{i}}_{\mathrm{mi}}-\sigma_{2i}{\bar{E}}_{\mathrm{mi}}-\mu_{i}{\bar{E}}_{\mathrm{mi}}+\delta_{E_{m}\mathrm{ji}}{\bar{E}}_{\mathrm{mj}}-\delta_{E_{m}\mathrm{ij}}{\bar{E}}_{\mathrm{mi}}\\ \alpha_{i}{\bar{S}}_{i}-\beta_{i}\delta_{1i}{\bar{V}}_{i}{\bar{i}}_{i}-\beta_{\mathrm{mi}}\delta_{2i}{\bar{V}}_{i}{\bar{i}}_{\mathrm{mi}}-\mu_{i}{\bar{V}}_{i}+\delta_{\mathrm{Vji}}{\bar{V}}_{j}-\delta_{\mathrm{Vij}}{\bar{V}}_{i}\;\\ \sigma_{1i}{\bar{E}}_{i}-u_{2i}{\bar{i}}_{i}-\gamma_{1i}{\bar{i}}_{i}-\mu_{i}{\bar{i}}_{i}+\delta_{\mathrm{Iji}}{\bar{i}}_{j}-\delta_{\mathrm{Iij}}{\bar{i}}_{i}\\ \sigma_{2i}{\bar{E}}_{\mathrm{mi}}-u_{2i}{\bar{i}}_{\mathrm{mi}}-\gamma_{2i}{\bar{i}}_{\mathrm{mi}}-\mu_{i}{\bar{i}}_{\mathrm{mi}}+\delta_{i_{m}\mathrm{ji}}{\bar{i}}_{\mathrm{mj}}-\delta_{i_{m}\mathrm{ij}}{\bar{i}}_{\mathrm{mi}}\\ u_{2i}{\bar{i}}_{i}+u_{2i}{\bar{i}}_{\mathrm{mi}}-\gamma_{3i}{\bar{Q}}_{i}-\mu_{i}{\bar{Q}}_{i}+\delta_{\mathrm{Qji}}{\bar{Q}}_{j}-\delta_{\mathrm{Qij}}{\bar{Q}}_{i}\\ \gamma_{1i}{\bar{i}}_{i}+\gamma_{2i}{\bar{i}}_{\mathrm{mi}}+\gamma_{3i}{\bar{Q}}_{i}-\mu_{i}{\bar{R}}_{i}-\phi_{S}{\bar{R}}_{i}+\delta_{\mathrm{Rji}}{\bar{R}}_{j}-\delta_{\mathrm{Rij}}{\bar{R}}_{i}\\ B_{j}-\beta_{j}{\bar{S}}_{j}{\bar{i}}_{j}-\beta_{\mathrm{mj}}{\bar{S}}_{j}{\bar{i}}_{\mathrm{mj}}-\alpha_{j}{\bar{S}}_{j}-\mu_{j}{\bar{S}}_{j}+\delta_{\mathrm{ij}}{\bar{S}}_{i}-\delta_{\mathrm{ji}}{\bar{S}}_{j}+\phi_{S}{\bar{R}}_{j}\\ \beta_{j}{\bar{S}}_{j}{\bar{i}}_{j}+\beta_{j}\delta_{1j}{\bar{V}}_{j}{\bar{i}}_{j}-\sigma_{1j}{\bar{E}}_{j}-\mu_{j}{\bar{E}}_{j}+{\delta_{E}}_{\mathrm{ij}}{\bar{E}}_{i}-{\delta_{E}}_{\mathrm{ji}}{\bar{E}}_{j}\\ \beta_{\mathrm{mj}}{\bar{S}}_{j}{\bar{i}}_{\mathrm{mj}}+\beta_{\mathrm{mj}}\delta_{2j}{\bar{V}}_{j}{\bar{i}}_{\mathrm{mj}}-\sigma_{2j}{\bar{E}}_{\mathrm{mj}}-\mu_{j}{\bar{E}}_{\mathrm{mj}}+\delta_{E_{m}\mathrm{ij}}{\bar{E}}_{\mathrm{mi}}-\delta_{E_{m}\mathrm{ji}}{\bar{E}}_{\mathrm{mj}}\\ \alpha_{j}{\bar{S}}_{j}-\beta_{j}\delta_{1j}{\bar{V}}_{j}{\bar{i}}_{j}-\beta_{\mathrm{mj}}\delta_{2j}{\bar{V}}_{j}{\bar{i}}_{\mathrm{mj}}-\mu_{j}{\bar{V}}_{j}-\delta_{\mathrm{Vji}}{\bar{V}}_{j}+\delta_{\mathrm{Vij}}{\bar{V}}_{i}\\ \sigma_{1j}{\bar{E}}_{j}-u_{2j}{\bar{i}}_{j}-\gamma_{1j}{\bar{i}}_{j}-\mu_{j}{\bar{i}}_{j}+\delta_{\mathrm{Iij}}{\bar{i}}_{i}-\delta_{\mathrm{Iji}}{\bar{i}}_{j}\\ \sigma_{2j}{\bar{E}}_{\mathrm{mj}}-u_{2j}{\bar{i}}_{\mathrm{mj}}-\gamma_{2j}{\bar{i}}_{\mathrm{mj}}-\mu_{j}{\bar{i}}_{\mathrm{mj}}+\delta_{i_{m}\mathrm{ij}}{\bar{i}}_{\mathrm{mi}}-\delta_{i_{m}\mathrm{ji}}{\bar{i}}_{\mathrm{mj}}\\ u_{2j}{\bar{i}}_{j}+u_{2j}{\bar{i}}_{\mathrm{mj}}-\gamma_{3j}{\bar{Q}}_{j}-\mu_{j}{\bar{Q}}_{j}+\delta_{\mathrm{Qij}}{\bar{Q}}_{i}-\delta_{\mathrm{Qji}}{\bar{Q}}_{j}\\ \gamma_{1j}{\bar{i}}_{j}+\gamma_{2j}{\bar{i}}_{\mathrm{mj}}+\gamma_{3j}{\bar{Q}}_{j}-\mu_{j}{\bar{R}}_{j}-\phi_{S}{\bar{R}}_{j}+\delta_{\mathrm{Rij}}{\bar{R}}_{i}-\delta_{\mathrm{Rji}}{\bar{R}}_{j} \end{array}\right)$$

### 3.4 Sliding mode control in stochastic model

The following defines a different PI sliding surface (Sam et al., 2004) for each control input. $G_{S1},G_{S2},G_{S3},$ and $G_{S4}$ be matrices chosen to define the sliding surface for each control input $u_{1i}\left(T\right),u_{2i}\left(T\right),u_{1j}\left(T\right),$ and $u_{2j}\left(T\right)$.

(7) $$S_{1i}\left(T\right)=G_{S1}X\left(T\right)-\int_{0}^{T}G_{S1}\left(A+B_{1}K_{1}\right)X\left(S\right)\mathrm{ds}$$

(8) $$S_{2i}\left(T\right)=G_{S2}X\left(T\right)-\int_{0}^{T}G_{S2}\left(A+B_{2}K_{2}\right)X\left(S\right)\mathrm{ds}$$

(9) $$S_{1j}\left(T\right)=G_{S3}X\left(T\right)-\int_{0}^{T}G_{S3}\left(A+B_{3}K_{3}\right)X\left(S\right)\mathrm{ds}$$

(10) $$S_{2j}\left(T\right)=G_{S4}X\left(T\right)-\int_{0}^{T}G_{S4}\left(A+B_{4}K_{4}\right)X\left(S\right)\mathrm{ds}$$

with $G_{\mathrm{si}}\in R^{16\times 16},i=1,2,3,4$ and $K_{i}\in R^{1\times 16}$ as constant row vectors for i=1,2,3,4. The matrix $K_{i}$ is designed to satisfy the stability condition $\lambda \left(A+B_{i}K_{i}\right)<0$, and the matrix $G_{\mathrm{si}}$ is chosen such that $G_{\mathrm{si}}B_{i}$ is not singular, ensuring that the defined sliding surface meets the desired stability requirements. The derivative of the sliding surface for each control input are as follows

$$dS_{1i}\left(T\right)=G_{S1}\mathrm{dX}\left(T\right)-G_{S1}\left(A+b_{1}K_{1}\right)X\left(T\right)\mathrm{dt}$$

$$dS_{2i}\left(T\right)=G_{S2}\mathrm{dX}\left(T\right)-G_{S2}\left(A+b_{2}K_{2}\right)X\left(T\right)\mathrm{dt}$$

$$dS_{1j}\left(T\right)=G_{S3}\mathrm{dX}\left(T\right)-G_{S3}\left(A+b_{3}K_{3}\right)X\left(T\right)\mathrm{dt}$$

$$dS_{2j}\left(T\right)=G_{S4}\mathrm{dX}\left(T\right)-G_{S4}\left(A+b_{4}K_{4}\right)X\left(T\right)\mathrm{dt}$$

substitute the stochastic model (6) into the derivative of the sliding surface, and since the expectation of $\mathrm{dW}\left(T\right)$ is zero, we obtain the following:

$$E\left\{dS_{1i}\left(T\right)\right\}=G_{S1}\left(\mathrm{AX}\left(T\right)+b_{1}u_{1i}\left(T\right)+b_{2}u_{2i}\left(T\right)+b_{3}u_{1j}\left(T\right)+b_{4}u_{2j}\left(T\right)+f\left(T\right)\right)\mathrm{dt}+G_{S1}S\left(X\left(T\right)\right)\mathrm{dW}\left(T\right)-G_{S1}\left(A+b_{1}K_{1}\right)X\left(T\right)\mathrm{dt}=0$$

$$E\left\{dS_{2i}\left(T\right)\right\}=G_{S2}\left(\mathrm{AX}\left(T\right)+b_{1}u_{1i}\left(T\right)+b_{2}u_{2i}\left(T\right)+b_{3}u_{1j}\left(T\right)+b_{4}u_{2j}\left(T\right)+f\left(T\right)\right)\mathrm{dt}-G_{S2}\left(A+b_{2}K_{2}\right)X\left(T\right)\mathrm{dt}=0$$

$$E\left\{dS_{1j}\left(T\right)\right\}=G_{S3}\left(\mathrm{AX}\left(T\right)+b_{1}u_{1j}\left(T\right)+b_{2}u_{2i}\left(T\right)+b_{3}u_{1j}\left(T\right)+b_{4}u_{2j}\left(T\right)+f\left(T\right)\right)\mathrm{dt}-G_{S3}\left(A+b_{3}K_{3}\right)X\left(T\right)\mathrm{dt}=0$$

$$E\left\{dS_{2j}\left(T\right)\right\}=G_{S4}\left(\mathrm{AX}\left(T\right)+b_{1}u_{1i}\left(T\right)+b_{2}u_{2i}\left(T\right)+b_{3}u_{1j}\left(T\right)+b_{4}u_{2j}\left(T\right)+f\left(T\right)\right)\mathrm{dt}-G_{S4}\left(A+b_{4}K_{4}\right)X\left(T\right)\mathrm{dt}=0$$

to determine $u_{1i}\left(T\right)$, assume $u_{2i}\left(T\right)=0$, $u_{1j}\left(T\right)=0$, $u_{2j}\left(T\right)=0$ we obtain

(11) $$u_{1i}\left(T\right)=K_{1}X\left(T\right)-{\left(G_{S1}B_{1}\right)}^{-1}G_{S1}f\left(T\right)$$

Similarly, following the steps above, we obtain

(12) $$u_{2i}\left(T\right)=K_{2}X\left(T\right)-{\left(G_{S2}B_{2}\right)}^{-1}G_{S2}f\left(T\right)$$

(13) $$u_{1j}\left(T\right)=K_{3}X\left(T\right)-{\left(G_{S3}B_{3}\right)}^{-1}G_{S3}f\left(T\right)$$

(14) $$u_{2j}\left(T\right)=K_{4}X\left(T\right)-{\left(G_{S4}B_{4}\right)}^{-1}G_{S4}f\left(T\right)$$

Substitute the control inputs (11)–(14) into the stochastic model (6),

(15) $$\mathrm{dX}\left(T\right)=\left(\left(A+B_{1}K_{1}+B_{2}K_{2}+B_{3}K_{3}+B_{4}K_{4}\right)X\left(T\right)+\left[i-B_{1}{\left(G_{S1}B_{1}\right)}^{-1}G_{S1}-B_{2}{\left(G_{S2}B_{2}\right)}^{-1}G_{S2}-B_{3}{\left(G_{S3}B_{3}\right)}^{-1}G_{S3}-B_{4}{\left(G_{S4}B_{4}\right)}^{-1}G_{S4}\right]f\left(T\right)\right)\mathrm{dt}+S\left(X\left(T\right)\right)\mathrm{dW}\left(T\right)$$

After determining the control rules, the next step is to perform a stability analysis of the system to ensure that the controlled system remains stable on the desired sliding surface. This stability analysis is crucial for evaluating the performance of the control system in handling uncertainties and stochastic disturbances.

### 3.5 Stability analysis

This subsection analyzes the stability of the closed-loop stochastic system using Lyapunov theory and Itô's Lemma, following the method proposed in (Moghadam and Kebriaei, 2020). The stability is analyzed based on the sliding surface introduced in Section 3.4, which includes a proportional-integral (PI) structure to enhance robustness under mismatched uncertainties. Let the Lyapunov candidate function be defined as $V\left(X\left(T\right)\right)=X^{T}\Psi X$, where $\Psi =\Psi^{T}>0$ is a symmetric positive definite matrix. By applying Itô's Lemma and computing the infinitesimal generator becomes:

$L\left(V\left(X\left(T\right)\right)\right)=X^{T}\mathrm{QX}$, where $Q=2\Psi \left(A+\mathrm{BK}\right)+S^{T}\Psi S.$

If Q<0, then the expected value of the Lyapunov function decreases over time, which indicates stochastics stability.

Theorem 1 *Consider the stochastic system defined by the differential eq*. (6), *with the sliding surface specified in eqs*. (11), (12), (13), (14). *Suppose there exist a symmetric positive definite matrix*$\Psi =\Psi^{T}>0$*and constant matrices*G,K,*and*B, *such that the following Linear Matrix Inequality holds*:

$$Q=2\Psi \left(A+\mathrm{BK}\right)+S^{T}\mathrm{\Psi S}<0$$

then the closed-loop stochastic system described in eq. (15) is mean-square stable.

In the stability analysis of stochastic systems, it is important not only to ensure that the system remains stable in the sense of bounded stability but also to evaluate exponential stability to guarantee a more controlled and rapid response toward equilibrium. After confirming that the system meets the bounded stability condition based on Theorem 1, we can proceed further by evaluating the exponential stability of the stochastic system. The following theorem states that if the conditions in Theorem 1 are satisfied, then the considered stochastic system is almost surely exponentially stable.

Theorem 2 Consider the stochastic system (6) and the sliding surface defined in eqs. (11), (12), (13), (14). If the conditions of Theorem 1 are satisfied, then the system is almost surely exponentially stable.

These stability conditions guarantee that the system's trajectory converges to the sliding surface in both mean-square and almost sure senses. This analysis confirms that the proposed control scheme can maintain the system's state within a bounded region and ensure stable behavior despite stochastic perturbations. The simulation results presented in the following section further demonstrate the effectiveness of this approach in controlling disease transmission in high-mobility environments.

### 3.6 Numerical simulation

**Fig. 4:**
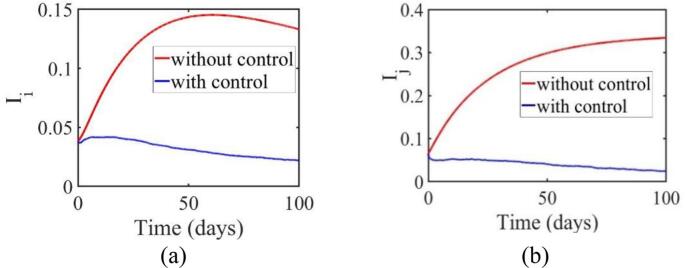
The dynamics of the original virus-infected population in the region of (a) Jakarta (b) West Java.

Using data from June 1 to August 31, 2021, parameter estimates were obtained using the Extended Kalman Filter (EKF), as shown in Table 2, and these parameters were used to simulate the infection dynamics of the populations in Jakarta and West Java. The simulation results, illustrated in Fig. 4, display the infected population trends over time for each region: (a) the original virus strain in Jakarta and (b) the original virus strain in West Java.

In both regions, the red curve represents infection levels without control, while the blue curve represents infection levels under sliding mode control (SMC). Without control, the infection counts show a sharp increase, peaking and stabilizing at higher levels in both regions. With control applied, the infected population remains at significantly lower levels throughout the simulation period.

**Fig. 5:**
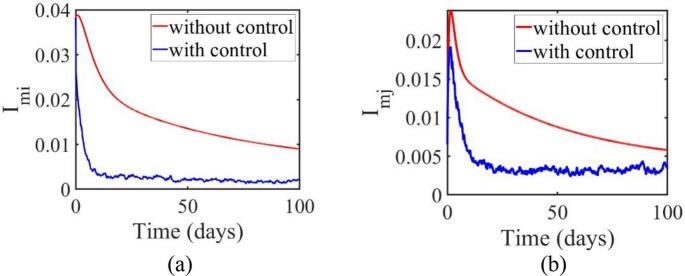
The dynamics of the Omicron-infected population in the region of (a) Jakarta (b) West Java.

In Jakarta (Fig. 4 (a)), the infection count without control rises sharply, reaching a peak around days 50–60, and then stabilizing. Under SMC, the infection level stays lower throughout the period, with a final count of 357,982 infected individuals compared to the 2,310,385 projected without control. In West Java (Fig. 4 (b)), a similar pattern is observed. Without control, infection numbers increase, stabilizing after peaking, while the application of SMC results in a reduced infection count, with a final count of 68,408 compared to the 5,852,872 projected without control. For the Omicron variant, the control measures show different outcomes. In Jakarta, infections drop from 91,228 to 32,902 with control. In West Java, infections decrease from 413,692 to 172,293 with control, indicating a moderate reduction in infection levels (Fig. 5). These results, as reflected in Table 3, highlight the significant impact of control measures in reducing infection levels compared to scenarios without control.

**Table 3:** Comparison of the average number of susceptible human populations infected with the original virus and the Omicron variant for 100 days

Condition	Without control	With control $u_{1}$ and $u_{2}$
Infected Population, Original Virus (Jakarta)	0.2054≈2,310,385	0.031821≈357,982
Infected Population, Omicron (Jakarta)	0.008109≈91,228	0.002925≈32,902
Infected Population, Original Virus (West Java)	0.1394≈5,852,872	0.001629≈68,408
Infected Population, Omicron (West Java)	0.00985≈413,692	0.004102≈172,293

**Fig. 6:**
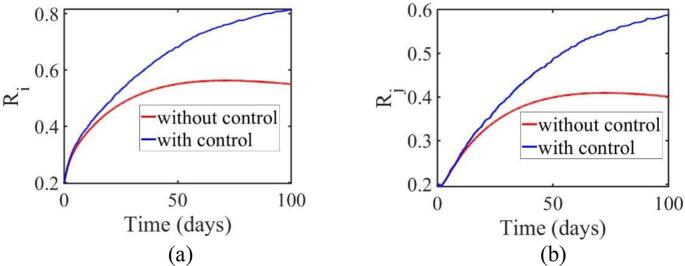
Dynamics confirmed to have recovered in areas (a) Jakarta (b) West Java.

Fig. 6 (a) shows the recovery trends in Jakarta. Without control, the recovery population rises gradually, reaching a level near 0.7 by day 100. With control, recoveries increase more rapidly, reaching 0.9 by day 100. In West Java (Fig. 6 (b)), a similar trend is observed. Without control, the recovery rate reaches 0.5 by day 100, while with control, the population reaches 0.55 by the end of the period.

**Fig. 7:**
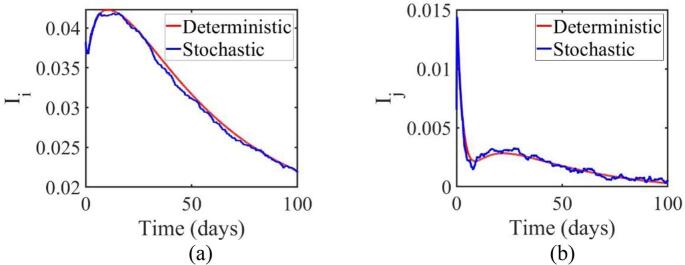
Comparison between deterministic and stochastic model curves in native virus populations (a) Jakarta (b) West Java.

In Fig. 7, the infected population for the original virus strain in Jakarta and West Java initially increases, peaking around day 14 in both (a) and (b), before gradually decreasing. Both the deterministic and stochastic models display similar overall trends, but the stochastic model exhibits greater fluctuations, especially after the peak infection period. This increased fluctuation in the stochastic model reflects real-world uncertainties that the deterministic model cannot fully capture. Although the stochastic model shows more variability, both models follow the same general pattern, with infection rates declining after reaching their peak.

**Fig. 8:**
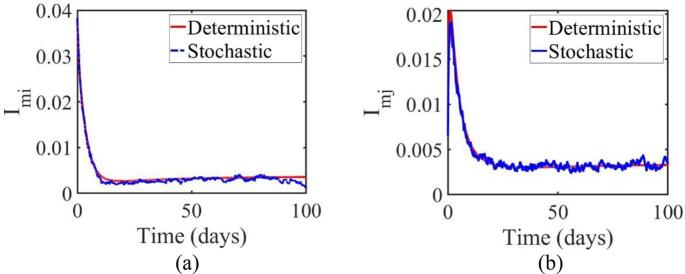
Comparison between deterministic and stochastic model curves in the Omicron variant population (a) Jakarta (b) West Java.

Both graphs also compare the deterministic and stochastic responses for $i_{\mathrm{mi}}$ and $i_{\mathrm{mj}}$ (Fig. 8). The trends in $i_{\mathrm{mi}}$ and $i_{\mathrm{mj}}$ decrease exponentially toward the end of the simulation period in both deterministic and stochastic models. The stochastic model displays minor fluctuations around the deterministic path due to stochastic noise present in the system. This indicates that the stochastic model realistically incorporates system uncertainties, providing a more comprehensive picture compared to the deterministic model, which does not account for external disturbances. For system stability, both models demonstrate long-term stability, as neither shows significant increases after reaching equilibrium. This suggests that the control strategies implemented are effective in stabilizing the observed variables $i_{\mathrm{mi}}$ and $i_{\mathrm{mj}}$, despite the presence of stochastic disturbances.

## 4 Discussion

This study presents a simulation-based framework for evaluating infection control strategies using sliding mode control (SMC) integrated with Extended Kalman Filter (EKF) estimation in a stochastic setting. Rather than proposing a directly deployable policy tool, the model demonstrates how theoretically robust control strategies, when combined with real-time estimation, can perform under uncertainty and partial observability, particularly in high-mobility regions such as Jakarta and West Java (Sutrisni et al., 2023). The simulation results show that SMC significantly reduces infection levels, especially for the original virus strain. In Jakarta, the number of infected individuals dropped from 2,310,385 to 357,982, achieving an 85 % reduction. In West Java, infections decreased from 5,852,872 to 68,408, amounting to a 98.6 % reduction. These findings are consistent with earlier on SMC for epidemic containment in deterministic settings (Jiao and Shen, 2020).

For the Omicron variant, control performance varies. In Jakarta, infections declined from 91,228 to 32,902, a reduction of approximately 64.8 %. In West Java, the infected population decreased from 413,000 to 172,293 under the same control strategy. These findings highlight that while SMC is effective for the original strain, additional adaptive strategies may be necessary for highly transmissible variants like Omicron, reflecting challenges previously reported for the Delta variant (Li and Guo, 2022). The effectiveness of interventions also varies by region. Vaccination appears particularly effective in high-density areas such as Jakarta, which supports earlier conclusions about the importance of population immunity and equitable vaccine access (Chi et al., 2022). In contrast, isolation alone appears less effective against Omicron in West Java, likely due to the variant's increased transmissibility and the difficulties associated with enforcing strict isolation in more dispersed populations (Djordjevic et al., 2023). These findings reinforce the need for hybrid strategies that combine vaccination and isolation, particularly in high-mobility settings.

Notably, the proposed control approach relies on state variables such as the number of susceptible and exposed individuals, which are not directly observable. To address this limitation, EKF is employed to estimate unmeasurable states from observable quantities, such as reported infection counts, vaccination data, and quarantine records. Observer-based estimation has been shown to improve control performance under partial observability in previous studies (Mehra et al., 2019).

The accuracy of the EKF-based estimates is validated through low Relative Root Mean Square Error (RRMSE) values: 2.96 % for Jakarta and 1.25 % for West Java. These results confirm the EKF's reliability in estimating real-time parameters and state dynamics, as also supported by prior findings (Hasan and Nasution, 2022). Accurate estimation is critical for enabling timely and effective control adjustments under evolving conditions. Furthermore, the stochastic modeling approach enhances realism by capturing random fluctuations in transmission due to behavioral changes and mobility, which aligns with previously established stochastic frameworks (Ghosh et al., 2023; Gunasekaran et al., 2023). In simulations, SMC consistently maintains a downward trend in infections, showcasing its robustness even in the presence of random disturbances.

In summary, this study contributes a robust simulation platform for assessing the effectiveness of control strategies in stochastic epidemic environments. While not immediately applicable as a public policy, the proposed framework supports the strategic evaluation of intervention measures under uncertainty. Future research could expand upon this by integrating predictive or adaptive control schemes to enhance flexibility and responsiveness in real-world outbreak scenarios (Mohammad and Kamrujjaman, 2025). This model assumes homogeneous mixing within each region and does not incorporate age-structured compartments, which could be addressed in future work.

## 5 Conclusions

This study developed a sliding mode control (SMC) framework combined with Extended Kalman Filter (EKF) estimation. The approach was applied to a stochastic epidemiological model that represents the transmission of infectious disease in regions with high levels of population mobility. The framework was designed to simulate and evaluate the effectiveness of vaccination and isolation strategies under uncertain conditions limited state observability, using real data from Jakarta and West Java as illustrative case studies.

The simulation results show that the proposed control framework significantly reduces infection levels. In Jakarta and West Java, reductions reached 84.45 % and 98.83 %, respectively, for the original strain, and 63.94 % and 58.35 % for the Omicron variant. These findings suggest that robust control methods, supported by real-time parameter estimation, can help maintain system stability and reduce transmission, even in complex and uncertain environments.

By comparing deterministic and stochastic models, the study highlights the importance of incorporating randomness into epidemiological modeling. The stochastic model captures fluctuations that cannot be addressed in deterministic settings and demonstrates how robust control can remain effective under variability and partial information. This framework offers a structured simulation-based approach for assessing epidemic control strategies, with potential applications in real-world planning as data assimilation and surveillance technologies continue to advance.

## CRediT authorship contribution statement

**Dewi Suhika:** Software, Resources, Methodology, Formal analysis, Conceptualization, Writing – review & editing, Writing – original draft. **Roberd Saragih:** Validation, Supervision, Methodology, Conceptualization, Writing – review & editing, Writing – original draft. **Dewi Handayani:** Validation, Investigation, Formal analysis, Writing – review & editing. **Mochamad Apri:** Validation, Methodology, Writing – review & editing.

## Declaration of competing interest

The authors declare that they have no known competing financial interests or personal relationships that could have appeared to influence the work reported in this paper.

## Data Availability

All data used in this study are available on the official websites: https://corona.jakarta.go.id/id for Jakarta and https://pikobar.jabarprov.go.id/ for West Java.

## Appendix A Supplementary data

Supplementary material Image 1
