# Supplementary material for: Sliding mode control with stochastic modeling and mobility interaction for managing epidemic spread in high-population regions
Source: Parasite Epidemiol Control. 2025 Jun 16;30:e00439. doi: 10.1016/j.parepi.2025.e00439 (PMC12221646; doi:10.1016/j.parepi.2025.e00439)
Supplement: Supplementary file 1 — Supplementary material [file mmc1.docx]

The first step in constructing a Stochastic Differential Equation (SDE) model is to identify all possible interactions that cause changes in the population or subpopulation sizes. Each change has a probability within a specific time step $\Delta t$, as presented in Table A. In this model, there are 60 possible changes within a $\Delta t$. The values 1, 0, and -1 in $\left( \Delta X \right)_{i}$ represent an increase, no change, and a decrease, respectively. These changes are referred to as transition changes, and each change has its own probability.

Note that from the model formulation (2), it has been explained that the population birth rate $b_{i}$ can increase the susceptible individual compartment $\bar{S}$, but does not affect other compartments. Assume that within the time interval $\Delta t$, $\bar{S}\to\bar{S}+1$. Thus, the transition vector from the change in $b_{i}$​ is:

$${\left( \Delta X \right)_{1}=\left( \begin{matrix} 1 & 0 & 0 & 0 & 0 & 0 & 0 & 0 & 0 & 0 & 0 & 0 & 0 & 0 & 0 0 \end{matrix} \right)}^{T}$$

The transition probability for the time interval $\Delta t$ is $p_{1}=b_{i}\Delta t$, which represents the probability of births entering the $\bar{S}$ population during the interval $\Delta t$. Meanwhile,

$$\left( \Delta X \right)_{2}=\left( \begin{matrix} -1 & 1 & 0 & 0 & 0 & 0 & 0 & 0 & 0 & 0 & 0 & 0 & 0 & 0 & 0 0 \end{matrix} \right)^{T}$$

represents the movement of individuals from the $\bar{S}$ population to the $\bar{E}$ population during the time interval $\Delta t$ with a probability of $p_{2}=\beta\bar{S}\bar{I}\Delta t$. Similarly, the transition vectors and transition probabilities for each component in model (2) are provided in Table A.

Table A. Transition probabilities are related to changes in the interacting population model.

| $\boldsymbol{i}$ | **Transition Vector** | **Transition Probability**$\left( \boldsymbol{p}_{\boldsymbol{i}} \right)$ |
| --- | --- | --- |
| 1 | $\left( \begin{matrix} 1 & 0 & 0 & 0 & 0 & 0 & 0 & 0 & 0 & 0 & 0 & 0 & 0 & 0 & 0 0 \end{matrix} \right)^{T}$ | $b_{i}\Delta t$ |
| 2 | $\left( \begin{matrix} -1 & 1 & 0 & 0 & 0 & 0 & 0 & 0 & 0 & 0 & 0 & 0 & 0 & 0 & 0 0 \end{matrix} \right)^{T}$ | $\beta_{i}\bar{S}_{i}\bar{I}_{i}\Delta t$ |
| 3 | $\left( -\begin{matrix} 1 & 0 & 1 & 0 & 0 & 0 & 0 & 0 & 0 & 0 & 0 & 0 & 0 & 0 & 0 0 \end{matrix} \right)^{T}$ | $\beta_{mi}\bar{S}_{i}\bar{I}_{mi}\Delta t$ |
| 4 | $\left( -\begin{matrix} 1 & 0 & 0 & 1 & 0 & 0 & 0 & 0 & 0 & 0 & 0 & 0 & 0 & 0 & 0 0 \end{matrix} \right)^{T}$ | $\alpha_{i}\bar{S}_{i}\Delta t$ |
| 5 | $\left( \begin{matrix} -1 & 0 & 0 & 0 & 0 & 0 & 0 & 0 & 0 & 0 & 0 & 0 & 0 & 0 & 0 0 \end{matrix} \right)^{T}$ | $\mu_{i}\bar{S}_{i}\Delta t$ |
| 6 | $\left( \begin{matrix} 1 & 0 & 0 & 0 & 0 & 0 & 0 & 0 & -1 & 0 & 0 & 0 & 0 & 0 & 0 0 \end{matrix} \right)^{T}$ | $\delta_{ji}\bar{S}_{j}\Delta t$ |
| 7 | $\left( \begin{matrix} -1 & 0 & 0 & 0 & 0 & 0 & 0 & 0 & 1 & 0 & 0 & 0 & 0 & 0 & 0 0 \end{matrix} \right)^{T}$ | $\delta_{ij}\bar{S}_{i}\Delta t$ |
| 8 | $\left( \begin{matrix} 1 & 0 & 0 & 0 & 0 & 0 & 0 & -1 & 0 & 0 & 0 & 0 & 0 & 0 & 0 0 \end{matrix} \right)^{T}$ | $\phi_{S}\bar{R}_{i}\Delta t$ |
| 9 | $\left( \begin{matrix} 0 & 1 & 0 & -1 & 0 & 0 & 0 & 0 & 0 & 0 & 0 & 0 & 0 & 0 & 0 0 \end{matrix} \right)^{T}$ | $\beta_{i}\delta_{1i}\bar{V}_{i}\bar{I}_{i}\Delta t$ |
| 10 | $\left( \begin{matrix} 0 & -1 & 0 & 0 & 1 & 0 & 0 & 0 & 0 & 0 & 0 & 0 & 0 & 0 & 0 0 \end{matrix} \right)^{T}$ | $\sigma_{1i}\bar{E}_{i}\Delta t$ |
| 11 | $\left( \begin{matrix} 0 & -1 & 0 & 0 & 0 & 0 & 0 & 0 & 0 & 0 & 0 & 0 & 0 & 0 & 0 0 \end{matrix} \right)^{T}$ | $\mu_{i}\bar{E}_{i}\Delta t$ |
| 12 | $\left( \begin{matrix} 0 & 1 & 0 & 0 & 0 & 0 & 0 & 0 & 0 & -1 & 0 & 0 & 0 & 0 & 0 0 \end{matrix} \right)^{T}$ | ${\delta_{E}}_{ji}\bar{E}_{j}\Delta t$ |
| 13 | $\left( \begin{matrix} 0 & -1 & 0 & 0 & 0 & 0 & 0 & 0 & 0 & 1 & 0 & 0 & 0 & 0 & 0 0 \end{matrix} \right)^{T}$ | ${\delta_{E}}_{ij}\bar{E}_{i}\Delta t$ |
| 14 | $\left( \begin{matrix} 0 & 0 & 1 & -1 & 0 & 0 & 0 & 0 & 0 & 0 & 0 & 0 & 0 & 0 & 0 0 \end{matrix} \right)^{T}$ | $\beta_{mi}\delta_{2i}\bar{V}_{i}\bar{I}_{mi}\Delta t$ |
| 15 | $\left( \begin{matrix} 0 & 0 & -1 & 0 & 0 & 1 & 0 & 0 & 0 & 0 & 0 & 0 & 0 & 0 & 0 0 \end{matrix} \right)^{T}$ | $\sigma_{2i}\bar{E}_{mi}\Delta t$ |
| 16 | $\left( \begin{matrix} 0 & 0 & -1 & 0 & 0 & 0 & 0 & 0 & 0 & 0 & 0 & 0 & 0 & 0 & 0 0 \end{matrix} \right)^{T}$ | $\mu_{i}\bar{E}_{mi}\Delta t$ |
| 17 | $\left( \begin{matrix} 0 & 0 & 1 & 0 & 0 & 0 & 0 & 0 & 0 & 0 & -1 & 0 & 0 & 0 & 0 0 \end{matrix} \right)^{T}$ | $\delta_{E_{m}ji}\bar{E}_{mj}\Delta t$ |
| 18 | $\left( \begin{matrix} 0 & 0 & -1 & 0 & 0 & 0 & 0 & 0 & 0 & 0 & 1 & 0 & 0 & 0 & 0 0 \end{matrix} \right)^{T}$ | $\delta_{E_{m}ij}\bar{E}_{mi}\Delta t$ |
| 19 | $\left( \begin{matrix} 0 & 0 & 0 & -1 & 0 & 0 & 0 & 0 & 0 & 0 & 0 & 0 & 0 & 0 & 0 0 \end{matrix} \right)^{T}$ | $\mu_{i}\bar{V}_{i}\Delta t$ |
| 20 | $\left( \begin{matrix} 0 & 0 & 0 & 1 & 0 & 0 & 0 & 0 & 0 & 0 & 0 & -1 & 0 & 0 & 0 0 \end{matrix} \right)^{T}$ | $\delta_{Vji}\bar{V}_{j}\Delta t$ |
| 21 | $\left( \begin{matrix} 0 & 0 & 0 & -1 & 0 & 0 & 0 & 0 & 0 & 0 & 0 & 1 & 0 & 0 & 0 0 \end{matrix} \right)^{T}$ | $\delta_{Vij}\bar{V}_{i}\Delta t$ |
| 22 | $\left( \begin{matrix} 0 & 0 & 0 & 0 & -1 & 0 & 1 & 0 & 0 & 0 & 0 & 0 & 0 & 0 & 0 0 \end{matrix} \right)^{T}$ | $u_{2i}\bar{I}_{i}\Delta t$ |
| 23 | $\left( \begin{matrix} 0 & 0 & 0 & 0 & -1 & 0 & 0 & 1 & 0 & 0 & 0 & 0 & 0 & 0 & 0 0 \end{matrix} \right)^{T}$ | $\gamma_{1i}\bar{I}_{i}\Delta t$ |
| 24 | $\left( \begin{matrix} 0 & 0 & 0 & 0 & -1 & 0 & 0 & 0 & 0 & 0 & 0 & 0 & 0 & 0 & 0 0 \end{matrix} \right)^{T}$ | $\mu_{i}\bar{I}_{i}\Delta t$ |
| 25 | $\left( \begin{matrix} 0 & 0 & 0 & 0 & 1 & 0 & 0 & 0 & 0 & 0 & 0 & 0 & -1 & 0 & 0 0 \end{matrix} \right)^{T}$ | $\delta_{Iji}\bar{I}_{j}\Delta t$ |
| 26 | $\left( \begin{matrix} 0 & 0 & 0 & 0 & -1 & 0 & 0 & 0 & 0 & 0 & 0 & 0 & 1 & 0 & 0 0 \end{matrix} \right)^{T}$ | $\delta_{Iij}\bar{I}_{i}\Delta t$ |
| 27 | $\left( \begin{matrix} 0 & 0 & 0 & 0 & 0 & -1 & 1 & 0 & 0 & 0 & 0 & 0 & 0 & 0 & 0 0 \end{matrix} \right)^{T}$ | $u_{2i}\bar{I}_{mi}\Delta t$ |
| 28 | $\left( \begin{matrix} 0 & 0 & 0 & 0 & 0 & -1 & 0 & 1 & 0 & 0 & 0 & 0 & 0 & 0 & 0 0 \end{matrix} \right)^{T}$ | $\gamma_{2i}\bar{I}_{mi}\Delta t$ |
| 29 | $\left( \begin{matrix} 0 & 0 & 0 & 0 & 0 & -1 & 0 & 0 & 0 & 0 & 0 & 0 & 0 & 0 & 0 0 \end{matrix} \right)^{T}$ | $\mu_{i}\bar{I}_{mi}\Delta t$ |
| 30 | $\left( \begin{matrix} 0 & 0 & 0 & 0 & 0 & 1 & 0 & 0 & 0 & 0 & 0 & 0 & 0 & -1 & 0 0 \end{matrix} \right)^{T}$ | $\delta_{I_{m}ji}\bar{I}_{mj}\Delta t$ |
| 31 | $\left( \begin{matrix} 0 & 0 & 0 & 0 & 0 & -1 & 0 & 0 & 0 & 0 & 0 & 0 & 0 & 1 & 0 0 \end{matrix} \right)^{T}$ | $\delta_{I_{m}ij}\bar{I}_{mi}\Delta t$ |
| 32 | $\left( \begin{matrix} 0 & 0 & 0 & 0 & 0 & 0 & -1 & 1 & 0 & 0 & 0 & 0 & 0 & 0 & 0 0 \end{matrix} \right)^{T}$ | $\gamma_{3i}\bar{Q}_{i}\Delta t$ |
| 33 | $\left( \begin{matrix} 0 & 0 & 0 & 0 & 0 & 0 & -1 & 0 & 0 & 0 & 0 & 0 & 0 & 0 & 0 0 \end{matrix} \right)^{T}$ | $\mu_{i}\bar{Q}_{i}\Delta t$ |
| 34 | $\left( \begin{matrix} 0 & 0 & 0 & 0 & 0 & 0 & 1 & 0 & 0 & 0 & 0 & 0 & 0 & 0 & -1 0 \end{matrix} \right)^{T}$ | $\delta_{Qji}\bar{Q}_{j}\Delta t$ |
| 35 | $\left( \begin{matrix} 0 & 0 & 0 & 0 & 0 & 0 & -1 & 0 & 0 & 0 & 0 & 0 & 0 & 0 & 1 0 \end{matrix} \right)^{T}$ | $\delta_{Qij}\bar{Q}_{i}\Delta t$ |
| 36 | $\left( \begin{matrix} 0 & 0 & 0 & 0 & 0 & 0 & 0 & -1 & 0 & 0 & 0 & 0 & 0 & 0 & 0 0 \end{matrix} \right)^{T}$ | $\mu_{i}\bar{R}_{i}\Delta t$ |
| 37 | $\left( \begin{matrix} 0 & 0 & 0 & 0 & 0 & 0 & 0 & 1 & 0 & 0 & 0 & 0 & 0 & 0 & 0 -1 \end{matrix} \right)^{T}$ | $\delta_{Rji}\bar{R}_{j}\Delta t$ |
| 38 | $\left( \begin{matrix} 0 & 0 & 0 & 0 & 0 & 0 & 0 & -1 & 0 & 0 & 0 & 0 & 0 & 0 & 0 1 \end{matrix} \right)^{T}$ | $\delta_{Rij}\bar{R}_{i}\Delta t$ |
| 39 | $\left( \begin{matrix} 0 & 0 & 0 & 0 & 0 & 0 & 0 & 0 & 1 & 0 & 0 & 0 & 0 & 0 & 0 0 \end{matrix} \right)^{T}$ | $b_{j}\Delta t$ |
| 40 | $\left( \begin{matrix} 0 & 0 & 0 & 0 & 0 & 0 & 0 & 0 & -1 & 1 & 0 & 0 & 0 & 0 & 0 0 \end{matrix} \right)^{T}$ | $\beta_{j}\bar{S}_{j}\bar{I}_{j}\Delta t$ |
| 41 | $\left( \begin{matrix} 0 & 0 & 0 & 0 & 0 & 0 & 0 & 0 & -1 & 0 & 1 & 0 & 0 & 0 & 0 0 \end{matrix} \right)^{T}$ | $\beta_{mj}\bar{S}_{j}\bar{I}_{mj}\Delta t$ |
| 42 | $\left( \begin{matrix} 0 & 0 & 0 & 0 & 0 & 0 & 0 & 0 & -1 & 0 & 0 & 1 & 0 & 0 & 0 0 \end{matrix} \right)^{T}$ | $\alpha_{j}\bar{S}_{j}\Delta t$ |
| 43 | $\left( \begin{matrix} 0 & 0 & 0 & 0 & 0 & 0 & 0 & 0 & -1 & 0 & 0 & 0 & 0 & 0 & 0 0 \end{matrix} \right)^{T}$ | $\mu_{j}\bar{S}_{j}\Delta t$ |
| 44 | $\left( \begin{matrix} 0 & 0 & 0 & 0 & 0 & 0 & 0 & 0 & 1 & 0 & 0 & 0 & 0 & 0 & 0 -1 \end{matrix} \right)^{T}$ | $\phi_{S}\bar{R}_{j}\Delta t$ |
| 45 | $\left( \begin{matrix} 0 & 0 & 0 & 0 & 0 & 0 & 0 & 0 & 0 & 1 & 0 & -1 & 0 & 0 & 0 0 \end{matrix} \right)^{T}$ | $\beta_{j}\delta_{1j}\bar{V}_{j}\bar{I}_{j}\Delta t$ |
| 46 | $\left( \begin{matrix} 0 & 0 & 0 & 0 & 0 & 0 & 0 & 0 & 0 & -1 & 0 & 0 & 1 & 0 & 0 0 \end{matrix} \right)^{T}$ | $\sigma_{1j}\bar{E}_{j}\Delta t$ |
| 47 | $\left( \begin{matrix} 0 & 0 & 0 & 0 & 0 & 0 & 0 & 0 & 0 & -1 & 0 & 0 & 0 & 0 & 0 0 \end{matrix} \right)^{T}$ | $\mu_{j}\bar{E}_{j}\Delta t$ |
| 48 | $\left( \begin{matrix} 0 & 0 & 0 & 0 & 0 & 0 & 0 & 0 & 0 & 0 & 1 & -1 & 0 & 0 & 0 0 \end{matrix} \right)^{T}$ | $\beta_{mj}\delta_{2j}\bar{V}_{j}\bar{I}_{mj}\Delta t$ |
| 49 | $\left( \begin{matrix} 0 & 0 & 0 & 0 & 0 & 0 & 0 & 0 & 0 & 0 & -1 & 0 & 0 & 1 & 0 0 \end{matrix} \right)^{T}$ | $\sigma_{2j}\bar{E}_{mj}\Delta t$ |
| 50 | $\left( \begin{matrix} 0 & 0 & 0 & 0 & 0 & 0 & 0 & 0 & 0 & 0 & -1 & 0 & 0 & 0 & 0 0 \end{matrix} \right)^{T}$ | $\mu_{j}\bar{E}_{mj}\Delta t$ |
| 51 | $\left( \begin{matrix} 0 & 0 & 0 & 0 & 0 & 0 & 0 & 0 & 0 & 0 & 0 & -1 & 0 & 0 & 0 0 \end{matrix} \right)^{T}$ | $\mu_{j}\bar{V}_{j}\Delta t$ |
| 52 | $\left( \begin{matrix} 0 & 0 & 0 & 0 & 0 & 0 & 0 & 0 & 0 & 0 & 0 & 0 & -1 & 0 & 1 0 \end{matrix} \right)^{T}$ | $u_{2j}\bar{I}_{j}\Delta t$ |
| 53 | $\left( \begin{matrix} 0 & 0 & 0 & 0 & 0 & 0 & 0 & 0 & 0 & 0 & 0 & 0 & -1 & 0 & 0 1 \end{matrix} \right)^{T}$ | $\gamma_{1j}\bar{I}_{j}\Delta t$ |
| 54 | $\left( \begin{matrix} 0 & 0 & 0 & 0 & 0 & 0 & 0 & 0 & 0 & 0 & 0 & 0 & -1 & 0 & 0 0 \end{matrix} \right)^{T}$ | $\mu_{j}\bar{I}_{j}\Delta t$ |
| 55 | $\left( \begin{matrix} 0 & 0 & 0 & 0 & 0 & 0 & 0 & 0 & 0 & 0 & 0 & 0 & 0 & -1 & 1 0 \end{matrix} \right)^{T}$ | $u_{2j}\bar{I}_{mj}\Delta t$ |
| 56 | $\left( \begin{matrix} 0 & 0 & 0 & 0 & 0 & 0 & 0 & 0 & 0 & 0 & 0 & 0 & 0 & -1 & 0 1 \end{matrix} \right)^{T}$ | $\gamma_{2j}\bar{I}_{mj}\Delta t$ |
| 57 | $\left( \begin{matrix} 0 & 0 & 0 & 0 & 0 & 0 & 0 & 0 & 0 & 0 & 0 & 0 & 0 & -1 & 0 0 \end{matrix} \right)^{T}$ | $\mu_{j}\bar{I}_{mj}\Delta t$ |
| 58 | $\left( \begin{matrix} 0 & 0 & 0 & 0 & 0 & 0 & 0 & 0 & 0 & 0 & 0 & 0 & 0 & 0 & -1 1 \end{matrix} \right)^{T}$ | $\gamma_{3j}\bar{Q}_{j}\Delta t$ |
| 59 | $\left( \begin{matrix} 0 & 0 & 0 & 0 & 0 & 0 & 0 & 0 & 0 & 0 & 0 & 0 & 0 & 0 & -1 0 \end{matrix} \right)^{T}$ | $\mu_{j}\bar{Q}_{j}\Delta t$ |
| 60 | $\left( \begin{matrix} 0 & 0 & 0 & 0 & 0 & 0 & 0 & 0 & 0 & 0 & 0 & 0 & 0 & 0 & 0 -1 \end{matrix} \right)^{T}$ | $\mu_{j}\bar{R}_{j}\Delta t$ |

The expectation and covariance matrices are calculated based on Table A. The transition table is sequentially influenced by human births, vaccination rates, infection rates, death rates, and recovery rates. The expectation matrix can be calculated as $E\left( \Delta X \right)=\sum_{i=1}^{60} p_{i}\left( \Delta X \right)_{i}$, which is a vector. For a given $\Delta t$, the expectation can be expressed as follows.

$$E\left( \Delta X \right)=\sum_{i=1}^{60} p_{i}\left( \Delta X \right)_{i}=p_{1}\left( \Delta X \right)_{1}+\ldots+p_{60}\left( \Delta X \right)_{60}$$

$$\mu=\frac{E\left( \Delta X \right)}{\Delta t}=A= \left( \begin{aligned} b_{i}-\beta_{i}\bar{S}_{i}\bar{I}_{i}-\beta_{mi}\bar{S}_{i}\bar{I}_{mi}-\alpha_{i}\bar{S}_{i}-\mu_{i}\bar{S}_{i}+\delta_{ji}\bar{S}_{j}-\delta_{ij}\bar{S}_{i}+\phi_{S}\bar{R}_{i} \\ \beta_{i}\bar{S}_{i}\bar{I}_{i}+\beta_{i}\delta_{1i}\bar{V}_{i}\bar{I}_{i}-\sigma_{1i}\bar{E}_{i}-\mu_{i}\bar{E}_{i}+{\delta_{E}}_{ji}\bar{E}_{j}-{\delta_{E}}_{ij}\bar{E}_{i} \\ \beta_{mi}\bar{S}_{i}\bar{I}_{mi}+\beta_{mi}\delta_{2i}\bar{V}_{i}\bar{I}_{mi}-\sigma_{2i}\bar{E}_{mi}-\mu_{i}\bar{E}_{mi}+\delta_{E_{m}ji}\bar{E}_{mj}-\delta_{E_{m}ij}\bar{E}_{mi} \\ \alpha_{i}\bar{S}_{i}-\beta_{i}\delta_{1i}\bar{V}_{i}\bar{I}_{i}-\beta_{mi}\delta_{2i}\bar{V}_{i}\bar{I}_{mi}-\mu_{i}\bar{V}_{i}+\delta_{Vji}\bar{V}_{j}-\delta_{Vij}\bar{V}_{i} \\ \sigma_{1i}\bar{E}_{i}-u_{2i}\bar{I}_{i}-\gamma_{1i}\bar{I}_{i}-\mu_{i}\bar{I}_{i}+\delta_{Iji}\bar{I}_{j}-\delta_{Iij}\bar{I}_{i} \\ \sigma_{2i}\bar{E}_{mi}-u_{2i}\bar{I}_{mi}-\gamma_{2i}\bar{I}_{mi}-\mu_{i}\bar{I}_{mi}+\delta_{I_{m}ji}\bar{I}_{mj}-\delta_{I_{m}ij}\bar{I}_{mi} \\ u_{2i}\bar{I}_{i}+u_{2i}\bar{I}_{mi}-\gamma_{3i}\bar{Q}_{i}-\mu_{i}\bar{Q}_{i}+\delta_{Qji}\bar{Q}_{j}-\delta_{Qij}\bar{Q}_{i} \\ \gamma_{1i}\bar{I}_{i}+\gamma_{2i}\bar{I}_{mi}+\gamma_{3i}\bar{Q}_{i}-\mu_{i}\bar{R}_{i}-\phi_{S}\bar{R}_{i}+\delta_{Rji}\bar{R}_{j}-\delta_{Rij}\bar{R}_{i} \\ b_{j}-\beta_{j}\bar{S}_{j}\bar{I}_{j}-\beta_{mj}\bar{S}_{j}\bar{I}_{mj}-\alpha_{j}\bar{S}_{j}-\mu_{j}\bar{S}_{j}+\delta_{ij}\bar{S}_{i}-\delta_{ji}\bar{S}_{j}+\phi_{S}\bar{R}_{j} \\ \beta_{j}\bar{S}_{j}\bar{I}_{j}+\beta_{j}\delta_{1j}\bar{V}_{j}\bar{I}_{j}-\sigma_{1j}\bar{E}_{j}-\mu_{j}\bar{E}_{j}+{\delta_{E}}_{ij}\bar{E}_{i}-{\delta_{E}}_{ji}\bar{E}_{j} \\ \beta_{mj}\bar{S}_{j}\bar{I}_{mj}+\beta_{mj}\delta_{2j}\bar{V}_{j}\bar{I}_{mj}-\sigma_{2j}\bar{E}_{mj}-\mu_{j}\bar{E}_{mj}+\delta_{E_{m}ij}\bar{E}_{mi}-\delta_{E_{m}ji}\bar{E}_{mj} \\ \alpha_{j}\bar{S}_{j}-\beta_{j}\delta_{1j}\bar{V}_{j}\bar{I}_{j}-\beta_{mj}\delta_{2j}\bar{V}_{j}\bar{I}_{mj}-\mu_{j}\bar{V}_{j}-\delta_{Vji}\bar{V}_{j}+\delta_{Vij}\bar{V}_{i} \\ \sigma_{1j}\bar{E}_{j}-u_{2j}\bar{I}_{j}-\gamma_{1j}\bar{I}_{j}-\mu_{j}\bar{I}_{j}+\delta_{Iij}\bar{I}_{i}-\delta_{Iji}\bar{I}_{j} \\ \sigma_{2j}\bar{E}_{mj}-u_{2j}\bar{I}_{mj}-\gamma_{2j}\bar{I}_{mj}-\mu_{j}\bar{I}_{mj}+\delta_{I_{m}ij}\bar{I}_{mi}-\delta_{I_{m}ji}\bar{I}_{mj} \\ u_{2j}\bar{I}_{j}+u_{2j}\bar{I}_{mj}-\gamma_{3j}\bar{Q}_{j}-\mu_{j}\bar{Q}_{j}+\delta_{Qij}\bar{Q}_{i}-\delta_{Qji}\bar{Q}_{j} \\ \gamma_{1j}\bar{I}_{j}+\gamma_{2j}\bar{I}_{mj}+\gamma_{3j}\bar{Q}_{j}-\mu_{j}\bar{R}_{j}-\phi_{S}\bar{R}_{j}+\delta_{Rij}\bar{R}_{i}-\delta_{Rji}\bar{R}_{j} \end{aligned} \right)$$

Next, the covariance matrix is defined as follows:

$$\mathcal{V=}E\left[ \left( \Delta X \right)\left( \Delta X \right)^{T} \right]$$

$$=\sum_{i=1}^{60} p_{i}\left( \Delta X \right)_{i}{\left( \Delta X \right)_{i}}^{T}=p_{1}\left( \Delta X \right)_{1}\left( \Delta X \right)_{1}^{T}+{p_{2}\left( \Delta X \right)}_{2}\left( \Delta X \right)_{2}^{T}+\ldots+p_{60}\left( \Delta X \right)_{60}\left( \Delta X \right)_{60}^{T}$$

$=$ $\left( \begin{matrix} v_{1} & -p_{2} & -p_{3} & -p_{4} & 0 & 0 & 0 & -p_{8} & v_{2} & 0 & 0 & 0 & 0 & 0 & 0 & 0 \\ -p_{2} & v_{3} & 0 & -p_{9} & -p_{10} & 0 & 0 & 0 & 0 & v_{4} & 0 & 0 & 0 & 0 & 0 & 0 \\ -p_{3} & 0 & v_{5} & -p_{14} & 0 & -p_{15} & 0 & 0 & 0 & 0 & v_{6} & 0 & 0 & 0 & 0 & 0 \\ -p_{4} & -p_{9} & -p_{14} & v_{7} & 0 & 0 & 0 & 0 & 0 & 0 & 0 & v_{8} & 0 & 0 & 0 & 0 \\ 0 & -p_{10} & 0 & 0 & v_{9} & 0 & -p_{22} & -p_{23} & 0 & 0 & 0 & 0 & v_{10} & 0 & 0 & 0 \\ 0 & 0 & {-p}_{15} & 0 & 0 & v_{11} & -p_{27} & -p_{28} & 0 & 0 & 0 & 0 & 0 & v_{12} & 0 & 0 \\ 0 & 0 & 0 & 0 & -p_{22} & -p_{27} & v_{13} & -p_{32} & 0 & 0 & 0 & 0 & 0 & 0 & v_{14} & 0 \\ -p_{8} & 0 & 0 & 0 & -p_{23} & -p_{28} & -p_{32} & v_{15} & 0 & 0 & 0 & 0 & 0 & 0 & 0 & v_{16} \\ v_{2} & 0 & 0 & 0 & 0 & 0 & 0 & 0 & v_{18} & -p_{40} & -p_{41} & {-p}_{42} & 0 & 0 & 0 & p_{44} \\ 0 & v_{4} & 0 & 0 & 0 & 0 & 0 & 0 & -p_{40} & v_{20} & 0 & -p_{45} & -p_{46} & 0 & 0 & 0 \\ 0 & 0 & v_{6} & 0 & 0 & 0 & 0 & 0 & -p_{41} & 0 & v_{22} & {-p}_{48} & 0 & -p_{49} & 0 & 0 \\ 0 & 0 & 0 & v_{8} & 0 & 0 & 0 & 0 & -p_{42} & -p_{45} & -p_{48} & v_{24} & 0 & 0 & 0 & 0 \\ 0 & 0 & 0 & 0 & v_{10} & 0 & 0 & 0 & 0 & -p_{46} & 0 & 0 & v_{26} & 0 & -p_{52} & -p_{53} \\ 0 & 0 & 0 & 0 & 0 & v_{12} & 0 & 0 & 0 & 0 & -p_{49} & 0 & 0 & v_{28} & -p_{55} & -p_{56} \\ 0 & 0 & 0 & 0 & 0 & 0 & v_{14} & 0 & 0 & 0 & 0 & 0 & -p_{52} & -p_{55} & v_{30} & -p_{58} \\ 0 & 0 & 0 & 0 & 0 & 0 & 0 & v_{16} & -p_{44} & 0 & 0 & 0 & -p_{53} & -p_{56} & -p_{58} & v_{32} \end{matrix} \right)$

where

$v_{1}=p_{1}+p_{2}+p_{3}+p_{4}+p_{5}+p_{6}+p_{7}+p_{8}$

$v_{2}=-p_{6}-p_{7}$

$v_{3}=p_{2}+p_{9}+p_{10}+p_{11}+p_{12}+p_{13}$

$v_{4}=-p_{12}-p_{13}$

$v_{5}=p_{3}+p_{14}+p_{15}+p_{16}+p_{17}+p_{18}$

$v_{6}=-p_{17}-p_{18}$

$v_{7}=p_{4}+p_{9}+p_{14}+p_{19}+p_{20}+p_{21}$

$v_{8}=-p_{20}-p_{21}$

$v_{9}=p_{10}+p_{22}+p_{23}+p_{24}+p_{25}+p_{26}$

$v_{10}=-p_{25}-p_{26}$

$v_{11}=p_{15}+p_{27}+p_{28}+p_{29}+p_{30}+p_{31}$

$v_{12}=-p_{30}-p_{31}$

$v_{13}={p_{22}+p_{27}+p}_{32}+p_{33}+p_{34}+p_{35}$

$v_{14}=-p_{34}-p_{35}$

$v_{15}=p_{23}+p_{28}+p_{32}+p_{36}+p_{37}+p_{38}$

$v_{16}=-p_{37}-p_{38}$

$v_{18}=p_{6}+p_{7}+p_{39}+p_{40}+p_{41}+p_{42}+p_{43}+p_{44}$

$v_{20}=p_{12}+p_{13}+p_{40}+p_{45}+p_{46}+p_{47}$

$v_{22}=p_{17}+p_{18}+p_{41}+p_{48}+p_{49}+p_{50}$

$v_{24}=p_{20}+p_{21}+p_{42}+p_{45}+p_{48}+p_{51}$

$v_{26}=p_{25}+p_{26}+p_{46}+p_{52}+p_{53}+p_{54}$

$v_{28}=p_{20}+p_{21}+p_{49}+p_{55}+p_{56}+p_{57}$

$v_{30}=p_{34}+p_{35}+p_{52}+p_{55}+p_{58}+p_{59}$

$v_{32}=p_{37}+p_{38}+p_{44}+p_{53}+p_{56}+p_{58}+p_{60}$

Define $Gg=\frac{E\left[ \left( \Delta X \right)\left( \Delta X \right)^{T} \right]}{\Delta t}$ as the weight matrix of disturbances. The disturbance weight matrix is obtained as follows:

$G_{g}=$ $\left( \begin{matrix} g_{1} & -g_{2} & -g_{3} & -g_{4} & 0 & 0 & 0 & -g_{5} & {-g}_{6} & 0 & 0 & 0 & 0 & 0 & 0 & 0 \\ -g_{2} & g_{7} & 0 & -g_{8} & -g_{10} & 0 & 0 & 0 & 0 & {-g}_{12} & 0 & 0 & 0 & 0 & 0 & 0 \\ -g_{3} & 0 & g_{9} & -g_{14} & 0 & -g_{15} & 0 & 0 & 0 & 0 & {-g}_{16} & 0 & 0 & 0 & 0 & 0 \\ -g_{4} & -g_{8} & -g_{14} & g_{11} & 0 & 0 & 0 & 0 & 0 & 0 & 0 & -g_{18} & 0 & 0 & 0 & 0 \\ 0 & -g_{10} & 0 & 0 & g_{13} & 0 & -g_{22} & -g_{23} & 0 & 0 & 0 & 0 & -g_{20} & 0 & 0 & 0 \\ 0 & 0 & {-g}_{15} & 0 & 0 & g_{17} & -g_{27} & -g_{28} & 0 & 0 & 0 & 0 & 0 & -g_{24} & 0 & 0 \\ 0 & 0 & 0 & 0 & -g_{22} & -g_{27} & g_{19} & -g_{32} & 0 & 0 & 0 & 0 & 0 & 0 & -g_{26} & 0 \\ -g_{5} & 0 & 0 & 0 & -g_{23} & -g_{28} & -g_{32} & g_{21} & 0 & 0 & 0 & 0 & 0 & 0 & 0 & -g_{30} \\ -g_{6} & 0 & 0 & 0 & 0 & 0 & 0 & 0 & g_{25} & -g_{40} & -g_{41} & {-g}_{42} & 0 & 0 & 0 & {-g}_{44} \\ 0 & -g_{12} & 0 & 0 & 0 & 0 & 0 & 0 & -g_{40} & g_{29} & 0 & -g_{45} & -g_{46} & 0 & 0 & 0 \\ 0 & 0 & -g_{16} & 0 & 0 & 0 & 0 & 0 & -g_{41} & 0 & g_{31} & {-g}_{48} & 0 & -g_{49} & 0 & 0 \\ 0 & 0 & 0 & -g_{18} & 0 & 0 & 0 & 0 & -g_{42} & -g_{45} & -g_{48} & g_{33} & 0 & 0 & 0 & 0 \\ 0 & 0 & 0 & 0 & -g_{20} & 0 & 0 & 0 & 0 & -g_{46} & 0 & 0 & g_{34} & 0 & -g_{52} & -g_{53} \\ 0 & 0 & 0 & 0 & 0 & -g_{24} & 0 & 0 & 0 & 0 & -g_{49} & 0 & 0 & g_{35} & -g_{55} & -g_{56} \\ 0 & 0 & 0 & 0 & 0 & 0 & -g_{26} & 0 & 0 & 0 & 0 & 0 & -g_{52} & -g_{55} & g_{36} & -g_{58} \\ 0 & 0 & 0 & 0 & 0 & 0 & 0 & -g_{30} & -g_{44} & 0 & 0 & 0 & -g_{53} & -g_{56} & -g_{58} & g_{37} \end{matrix} \right)$

where

$g_{1}=b_{i}+\beta_{i}\bar{S}_{i}\bar{I}_{i}+\beta_{mi}\bar{S}_{i}\bar{I}_{mi}+\alpha_{i}\bar{S}_{i}+\mu_{i}\bar{S}_{i}+\delta_{ji}\bar{S}_{j}+\delta_{ij}\bar{S}_{i}+\phi_{S}\bar{R}_{i}$

$g_{2}=\beta_{i}\bar{S}_{i}\bar{I}_{i}$

$g_{3}=\beta_{mi}\bar{S}_{i}\bar{I}_{mi}$

$g_{4}=\alpha_{i}\bar{S}_{i}$

$g_{5}=\phi_{S}\bar{R}_{i}$

$g_{6}=\delta_{ji}\bar{S}_{j}+\delta_{ij}\bar{S}_{i}$

$g_{7}=\beta_{i}\bar{S}_{i}\bar{I}_{i}+\beta_{i}\delta_{1i}\bar{V}_{i}\bar{I}_{i}+\sigma_{1i}\bar{E}_{i}+\mu_{i}\bar{E}_{i}+{\delta_{E}}_{ji}\bar{E}_{j}+{\delta_{E}}_{ij}\bar{E}_{i}$

$g_{8}=\beta_{i}\delta_{1i}\bar{V}_{i}\bar{I}_{i}$

$g_{9}=\beta_{mi}\bar{S}_{i}\bar{I}_{mi}+\beta_{mi}\delta_{2i}\bar{V}_{i}\bar{I}_{mi}+\sigma_{2i}\bar{E}_{mi}+\mu_{i}\bar{E}_{mi}+\delta_{E_{m}ji}\bar{E}_{mj}+\delta_{E_{m}ij}\bar{E}_{mi}$

$g_{10}=\sigma_{1i}\bar{E}_{i}$

$g_{11}=\alpha_{i}\bar{S}_{i}+\beta_{i}\delta_{1i}\bar{V}_{i}\bar{I}_{i}+\beta_{mi}\delta_{2i}\bar{V}_{i}\bar{I}_{mi}+\mu_{i}\bar{V}_{i}+\delta_{Vji}\bar{V}_{j}+\delta_{Vij}\bar{V}_{i}$

$g_{12}={\delta_{E}}_{ji}\bar{E}_{j}+{\delta_{E}}_{ij}\bar{E}_{i}$

$g_{13}=\sigma_{1i}\bar{E}_{i}+u_{2i}\bar{I}_{i}+\gamma_{1i}\bar{I}_{i}+\mu_{i}\bar{I}_{i}+\delta_{Iji}\bar{I}_{j}+\delta_{Iij}\bar{I}_{i}$

$g_{14}=\beta_{mi}\delta_{2i}\bar{V}_{i}\bar{I}_{mi}$

$g_{15}=\sigma_{2i}\bar{E}_{mi}$

$g_{16}=\delta_{E_{m}ji}\bar{E}_{mj}+\delta_{E_{m}ij}\bar{E}_{mi}$

$g_{17}=\sigma_{2i}\bar{E}_{mi}+u_{2i}\bar{I}_{mi}+\gamma_{2i}\bar{I}_{mi}+\mu_{i}\bar{I}_{mi}+\delta_{I_{m}ji}\bar{I}_{mj}+\delta_{I_{m}ij}\bar{I}_{mi}$

$g_{18}=\delta_{Vji}\bar{V}_{j}+\delta_{Vij}\bar{V}_{i}$

$g_{19}=u_{2i}\bar{I}_{i}+u_{2i}\bar{I}_{mi}+\gamma_{3i}\bar{Q}_{i}+\mu_{i}\bar{Q}_{i}+\delta_{Qji}\bar{Q}_{j}+\delta_{Qij}\bar{Q}_{i}$

$g_{20}=\delta_{Iji}\bar{I}_{j}+\delta_{Iij}\bar{I}_{i}$

$g_{21}=\gamma_{1i}\bar{I}_{i}+\gamma_{2i}\bar{I}_{mi}+\gamma_{3i}\bar{Q}_{i}+\mu_{i}\bar{R}_{i}+\delta_{Rji}\bar{R}_{j}+\delta_{Rij}\bar{R}_{i}$

$g_{22}=u_{2i}\bar{I}_{i}$

$g_{23}=\gamma_{1i}\bar{I}_{i}$

$g_{24}=\delta_{I_{m}ji}\bar{I}_{mj}+\delta_{I_{m}ij}\bar{I}_{mi}$

$g_{25}=\delta_{ji}\bar{S}_{j}+\delta_{ij}\bar{S}_{i}+b_{j}+\beta_{j}\bar{S}_{j}\bar{I}_{j}+\beta_{mj}\bar{S}_{j}\bar{I}_{mj}+\alpha_{j}\bar{S}_{j}+\mu_{j}\bar{S}_{j}+\phi_{S}\bar{R}_{j}$

$g_{26}=\delta_{Qji}\bar{Q}_{j}+\delta_{Qij}\bar{Q}_{i}$

$g_{27}=u_{2i}\bar{I}_{mi}$

$g_{28}=\gamma_{2i}\bar{I}_{mi}$

$g_{29}={\delta_{E}}_{ji}\bar{E}_{j}+{\delta_{E}}_{ij}\bar{E}_{i}+\beta_{j}\bar{S}_{j}\bar{I}_{j}+\beta_{j}\delta_{1j}\bar{V}_{j}\bar{I}_{j}+\sigma_{1j}\bar{E}_{j}+\mu_{j}\bar{E}_{j}$

$g_{30}=\delta_{Rji}\bar{R}_{j}+\delta_{Rij}\bar{R}_{i}$

$g_{31}=\delta_{E_{m}ji}\bar{E}_{mj}+\delta_{E_{m}ij}\bar{E}_{mi}+\beta_{mj}\bar{S}_{j}\bar{I}_{mj}+\beta_{mj}\delta_{2j}\bar{V}_{j}\bar{I}_{mj}+\sigma_{2j}\bar{E}_{mj}+\mu_{j}\bar{E}_{mj}$

$g_{32}=\gamma_{3i}\bar{Q}_{i}$

$g_{33}=\delta_{Vji}\bar{V}_{j}+\delta_{Vij}\bar{V}_{i}+\alpha_{j}\bar{S}_{j}+\beta_{j}\delta_{1j}\bar{V}_{j}\bar{I}_{j}+\beta_{mj}\delta_{2j}\bar{V}_{j}\bar{I}_{mj}+\mu_{j}\bar{V}_{j}$

$g_{34}=\delta_{Iji}\bar{I}_{j}+\delta_{Iij}\bar{I}_{i}+\sigma_{1j}\bar{E}_{j}+u_{2j}\bar{I}_{j}+\gamma_{1j}\bar{I}_{j}+\mu_{j}\bar{I}_{j}$

$g_{35}=\delta_{Vji}\bar{V}_{j}+\delta_{Vij}\bar{V}_{i}+\sigma_{2j}\bar{E}_{mj}+u_{2j}\bar{I}_{mj}+\gamma_{2j}\bar{I}_{mj}+\mu_{j}\bar{I}_{mj}$

$g_{36}=\delta_{Qji}\bar{Q}_{j}+\delta_{Qij}\bar{Q}_{i}+u_{2j}\bar{I}_{j}+u_{2j}\bar{I}_{mj}+\gamma_{3j}\bar{Q}_{j}+\mu_{j}\bar{Q}_{j}$

$g_{37}=\delta_{Rji}\bar{R}_{j}+\delta_{Rij}\bar{R}_{i}+\phi_{S}\bar{R}_{j}+\gamma_{1j}\bar{I}_{j}+\gamma_{2j}\bar{I}_{mj}+\gamma_{3j}\bar{Q}_{j}+\mu_{j}\bar{R}_{j}$

$g_{40}=\beta_{j}\bar{S}_{j}\bar{I}_{j}$

$g_{41}=\beta_{mj}\bar{S}_{j}\bar{I}_{mj}$

$g_{42}=\alpha_{j}\bar{S}_{j}$

$g_{44}=\phi_{S}\bar{R}_{j}$

$g_{45}=\beta_{j}\delta_{1j}\bar{V}_{j}\bar{I}_{j}$

$g_{46}=\sigma_{1j}\bar{E}_{j}$

$g_{48}=\beta_{mj}\delta_{2j}\bar{V}_{j}\bar{I}_{mj}$

$g_{49}=\sigma_{2j}\bar{E}_{mj}$

$g_{52}=u_{2j}\bar{I}_{j}$

$g_{53}=\gamma_{1j}\bar{I}_{j}$

$g_{55}=u_{2j}\bar{I}_{mj}$

$g_{56}=\gamma_{2j}\bar{I}_{mj}$

$g_{58}=\gamma_{3j}\bar{Q}_{j}$
